# Sub-strains of *Drosophila* Canton-S differ markedly in their locomotor behavior

**DOI:** 10.12688/f1000research.4263.2

**Published:** 2015-04-21

**Authors:** Julien Colomb, Björn Brembs

**Affiliations:** 1 Institute of Biology – Neurobiology, Freie Universität, Berlin, Germany; 2 Institute of Zoology – Neurogenetics, Universität Regensburg, Regensburg, Germany

**Keywords:** Genetic background, Buridan's paradigm, walking behavior, wild-type

## Abstract

We collected five sub-strains of the standard laboratory wild-type *Drosophila* *melanogaster *Canton Special (CS) and analyzed their walking behavior in Buridan's paradigm using the CeTrAn software. According to twelve different aspects of their behavior, the sub-strains fit into three groups. The group separation appeared not to be correlated with the origin of the stocks. We conclude that founder effects but not laboratory selection likely influenced the gene pool of the sub-strains. The flies’ stripe fixation was the parameter that varied most. Our results suggest that differences in the genome of laboratory stocks can render comparisons between nominally identical wild-type stocks meaningless. A single source for control strains may settle this problem.

## Introduction

In our quest for understanding gene function, we commonly manipulate gene expression and compare the phenotypes of the manipulated versus control organisms. For technical reasons and to facilitate comparison as well as reproducibility between different experiments, a limited number of control strains have been established in most model organisms. For instance, the C57BL, 129 and FVB strains are commonly used in mouse studies; N2 is the common control strain used in *Caenorhabditis elegans*; and Canton-Special (CS) is one of the most-used wild-type strains in *Drosophila melanogaster* genetics studies. The CS stock was established by C. B. Bridges ^1^ and chosen because of its low mutation rate. S. Benzer introduced CS to what was to become neurogenetics in his landmark study in 1967 ^2^, because of its strong fast-phototaxis response. The strain has been used as a control in neurogenetics studies ever since.

With time and reproductive isolation, populations of laboratory control strains can diverge, in spite of ideal breeding conditions and seemingly little selective pressure. Several studies comparing the behavior of sub-strains of mice showed that their behavior differs ^3^. For instance, Paylor and colleagues measured that one sub-strain of C57 mice showed a higher startle amplitude after tactile stimulation than another ^4^. Similarly, the behavior of different N2 *C. elegans* sub-strains was found to vary to a considerable extent ^5^.

In this study, we tested five different CS *Drosophila melanogaster* sub-strains in Buridan’s paradigm ^6– 8^), where flies walk between two stripes on a platform surrounded by a water moat. We could separate the CS sub-strains into three groups according to their behavior during the experiment. In addition, we found that the between-strain variability in the stripe fixation score is particularly high. We discuss possible solutions to prevent sub-strain related problems.

## Materials and methods

### Fly care

Flies were kept in vials (68 ml, Art.-Nr. 217101, Greiner Bio-One GmbH, Maybachstr.2, 72636 Frickenhausen) in a controlled density on standard cornmeal/molasses medium ^9^ at 25°C in a 12 h:12 h dark/light cycle for one generation before being tested. Flies were collected 0–1 day after hatching and put in new food vials for one day. Approximately ten female flies (N=11–12 in each group) were then CO _2_-anaesthetized and their wings were cut with surgical scissors at two thirds of their length, before being taken back to their vial to recover overnight. They were then captured individually using a fly aspirator and put in the experimental setup to be tested.

### Fly strains

Five sub-strains of CS wild-type *Drosophila melanogaster* were collected in the lab from 2008 to 2011. Troy Zars took his CS_TZ stock to Columbia, MO, USA in 2002 when leaving Martin Heisenberg’s lab in Würzburg, Germany. It arrived in our laboratory in Berlin in 2008. The CS_TP stock was separated from Tim Tully’s strain in Waltham, MA, USA in 1992 and moved to Paris, France. The CS_JC stock was derived from the CS_TP stock in 2007 when one of the authors (JC) was in Thomas Préat’s lab. Both strains (TP and JC) arrived in Berlin in 2009. The CS_BS stock was separated by Bruno van Swinderen from Ralf Greenspan’s stock in San Diego, CA, USA in 1999 and brought to Brisbane, Australia. From there it arrived in Berlin in 2008. Finally, Henrike Scholz received her stock from Ulrike Heberlein in San Francisco, CA, USA. The CS_HS strain arrived in Berlin in 2007. Flies of all strains were kept at 18°C until being tested in 2012 and 2013.

### Buridan’s paradigm

Experimental details are described in detail elsewhere ^7^. Briefly, two black stripes producing 11° wide landmarks were positioned 293 mm from the center of a platform with a diameter of 117 mm, surrounded by water and illuminated with bright white light from behind. The centroid position of the fly was recorded via custom software (BuriTrack, http://buridan.sourceforge.net). If flies jumped from the platform, they were taken back to the platform with a brush and the tracker was reinitialized. Each data file represents five minutes of uninterrupted walking. We measured two replicates of the same five sub-strains in consecutive years, 2012 and 2013.

For more than three decades, experiments in Buridan’s paradigm demonstrated that wild-type flies typically walk back and forth between the landmarks. We performed 5 minute long walking experiments with five different wild type Canton S (CS) sub-strains: CS_TP, CS_TZ, CS_JC, CS_BS and CS_HS. The locomotion parameters we calculated can be divided into three broad categories: temporal (activity/pause structure), spatial (stripe fixation, thigmotaxis, trajectory straightness) and mixed (speed, number of walks between stripes, distance travelled) measures (ref. 7, Table 1).

**Table 1.** Brief description of the twelve parameters calculated from the trajectory of the flies used in this paper. A more detailed description is available at http://dx.doi.org/10.6084/m9.figshare.844624.

Parameter	Description
Median speed	Median of the speed of the animal while walking
Mean distance travelled per min.	Distance travelled during the experiment divided by the duration of the experiment
Turning angle	Median of the angle difference between two movements
Meander	Median of the turning angle divided by instantaneous speed
Thigmotaxis while moving	Proportion of time spent on the edge of the platform versus the center of the platform (equal surfaces) while moving
Thigmotaxis while sitting	Proportion of time spent on the edge of the platform versus the center of the platform (equal surfaces) while being immobile
Stripe deviation	Median deviation angle between walking direction and direction toward the stripes
Number of walks	Number of times a fly walks between the two stripes during the experiment
Number of pauses	Number of times a fly stopped walking for more than 1s during the experiment
Activity bouts duration	Median duration of activity phases
Pause length	Median duration of pauses
Total time active	Sum of the length of activity phases during the experiment

### Experimental differences between the replicates

The experiments in 2012 were done according to the previously published setup ^7^, while the 2013 experiments were performed in four new setups. In the new setups, illumination is slightly brighter (10–11 klx in the new setup, 7.5–8.5 klx in the old setup). We did not detect any difference in the temperature on the platforms (27°C for all machines). The platform was cleaned between flies in the 2012 replicate, while the platform was rotated between two tests in the 2013 replicate, and cleaned only after a series of five flies had been tested.

### Analysis

The data was analyzed using CeTrAn v.4 ( https://github.com/jcolomb/CeTrAn/releases/tag/v.4). Data with a mean distance travelled smaller than 50 mm/min was excluded to avoid outliers (2 data points were excluded in the second replicate, one for CS_JC and one for CS_HS).

Twelve different parameters were calculated ( Table 1) and a Principal Components Analysis (PCA) was performed to visualize the results and identify potential groupings of the sub-strains. The effects of genotype and replicate were analyzed with an ANOVA (in R) using the second principal component, since the first and the third components were not normally distributed (assessed with a Shapiro test). Transition plots and the stripe deviation plot have not been tested statistically.

### Data availability

Raw trajectory data (including outliers), the results of the CeTrAn analysis and the PCA result table are available on figshare: http://dx.doi.org/10.6084/m9.figshare.1014264.

## Results

Buridan raw data: Sub-strains of Drosophila Canton-S differ markedly in their locomotor behavior Raw data for the F1000 Research paper "Sub-strains of Drosophila Canton-S differ markedly in their locomotor behavior"
Code is CeTrAn 4.0 (on github)
F1000 Research article doi: 10.12688/f1000research.4263.1 Click here for additional data file. Copyright: © 2015 Colomb J and Brembs B 2015 Data associated with the article are available under the terms of the Creative Commons Zero "No rights reserved" data waiver (CC0 1.0 Public domain dedication).

**Figure 1.**
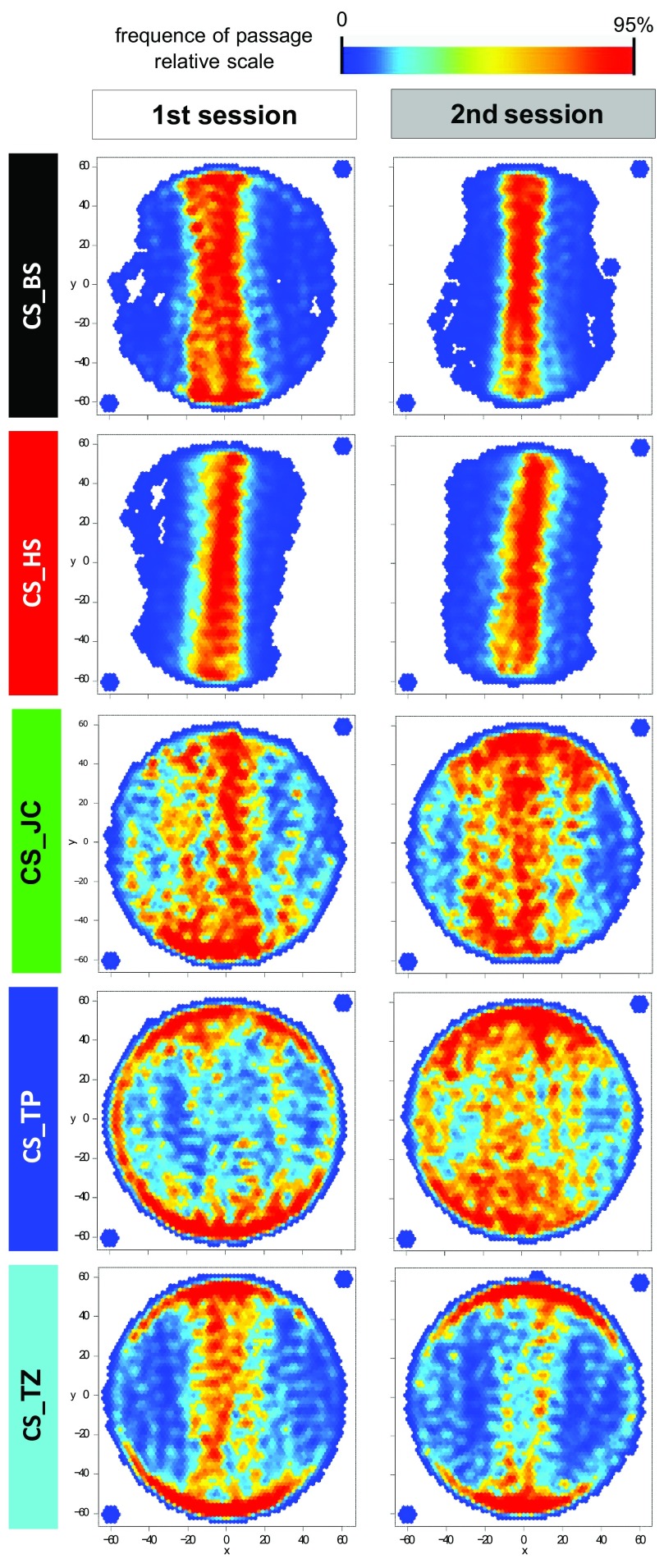
In transition plots, the behavior of each sub-strain looks different from the other strains and similar between the two experimental sessions. Transition plots represent the position of the fly on the platform, excluding the time when the fly was immobile. The scale is proportional, with red points meaning that the number of times the fly was in that position is at least 95% of the maximal score obtained for any position. A Gaussian smooth was applied to the resulting heat map. The two points outside the platform were added manually to assure orthogonal axes of the representation. Sample size is 11–12 for each plot.

In Buridan’s paradigm, wild-type flies typically walk back and forth between two inaccessible landmarks and their walking behavior is then analyzed. We performed 5 minutes long walking experiments with five different Canton S (CS) sub-strains: CS_TP, CS_TZ, CS_JC, CS_BS and CS_HS. We tested them in two replicates in two consecutive years using different hardware and under slightly varying experimental details (see Materials and Methods). The locomotion parameters that we calculated can be divided into three broad categories: temporal (activity/pause structure), spatial (stripe fixation, thigmotaxis, trajectory straightness) and mixed (speed, number of walks between stripes, distance travelled) measures (ref. 7, Table 1). Flies’ walking behavior was also visualized in transition plots, where the frequency of passage at each platform position is indicated by a heatmap. A distinction between sub-strains, which is consistent between the two replicates, can be seen in the visualization of this purely spatial parameter ( Figure 1).

**Figure 2.**
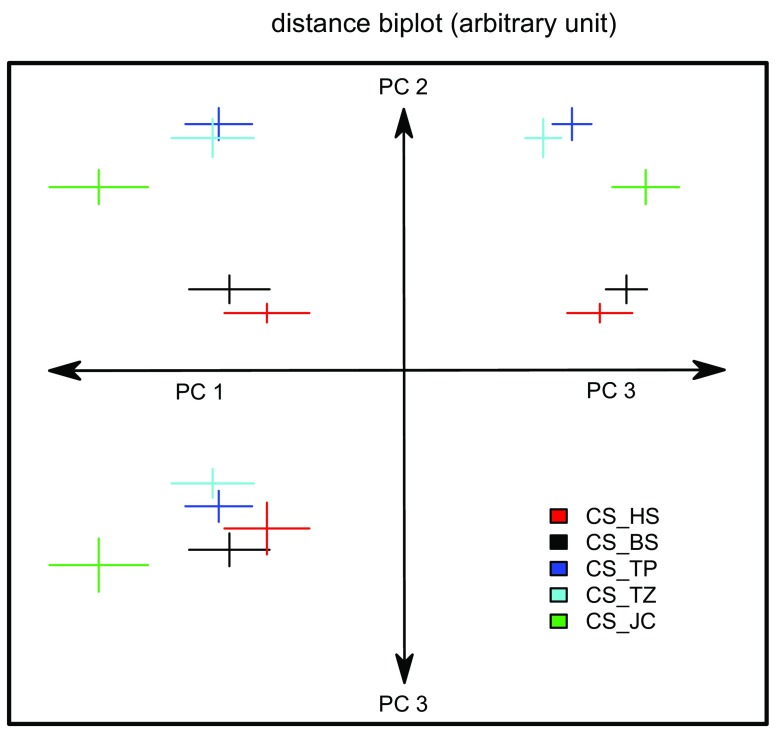
The CS sub-strains can be separated into three groups according to their overall behavior in Buridan’s paradigm. A PCA was performed over the 12 measured variables capturing the flies’ locomotion. The three first principal components are plotted against each other: from the center of the axes; PC1 to the left, PC2 up and PC3 down and to the right. Since units are arbitrary, they were not indicated. For each genotype, we represent the mean and standard error of the mean for the different PCs as a colored cross (data from the two replicates were pooled). The three groups are best visualized separately on the PC2-PC3 plot (upper-right), while PC2 is sufficient to separate the three groups statistically (see text). Sample size for each group is 23–28.

Using CeTrAn 4.0, we took twelve measurements of the flies’ walking behavior and analyzed them using PCA (for individual performance of each strain in each measurement, see supplementary materials). For simplicity of representation, we plotted the mean and standard error of the three first principal components, while pooling the replicates ( Figure 2). Since the first and third principal components were not normally distributed (Shapiro test), we performed an ANOVA for the second component with the fly sub-strains and the replicates as factors. This analysis demonstrated significant main effects of the sub-strain (F = 28.305, p<2e-16) and the replicate (F = 9.35, p<0.003), while there seems to be no sub-strain × replicate interaction (F value = 2.337, p = 0.059). A Tukey HSD post hoc test of the sub-strain effect confirmed the visual impression of the PCA grouping: CS_TZ and CS_TP together in one group, CS_BS and CS_HS together and CS_JC alone.

**Figure 3.**
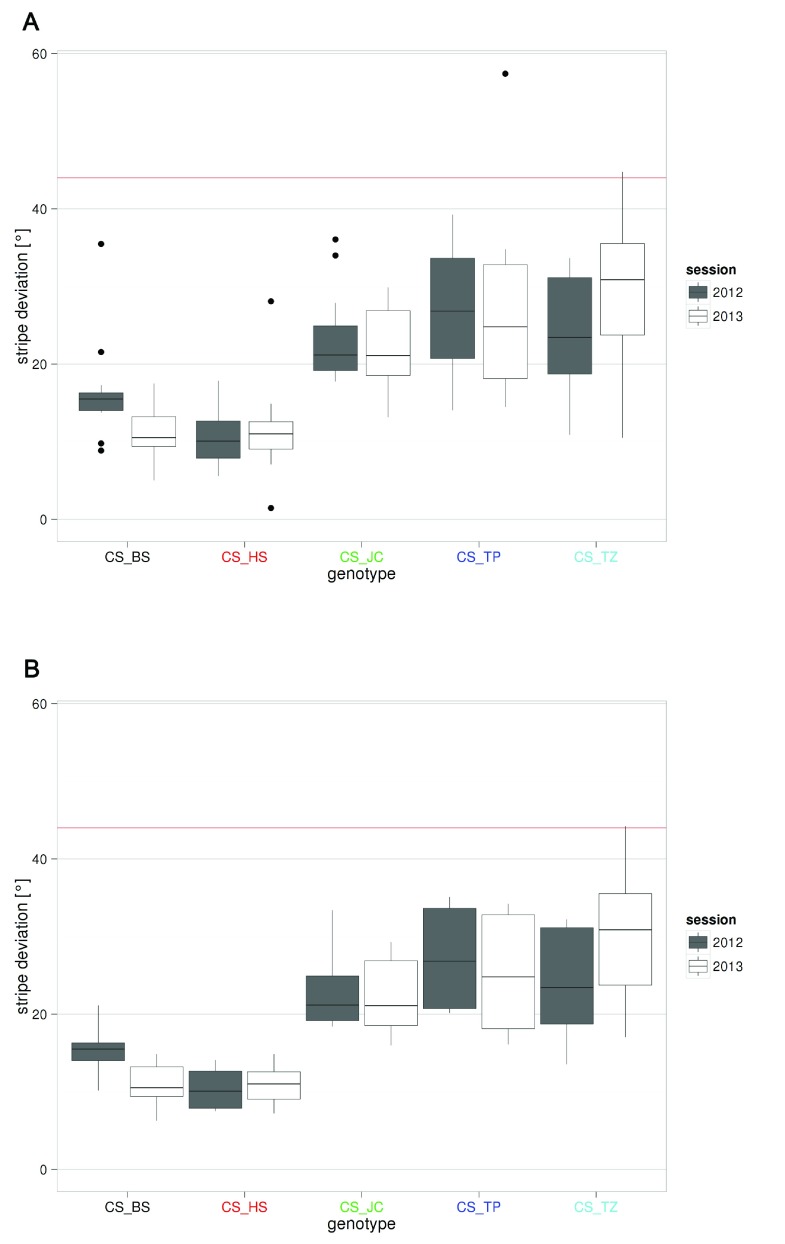
The different sub-strains show a large spectrum of values for the stripe deviation parameter. For every movement of the fly, the angle between its direction and the direction toward the stripes was calculated. The median of these angles was calculated for each fly, representing a quantification of stripe fixation by the fly. The value of each sub-strain in each session is depicted in boxplots: for each group, we represent the median, 25–75% quantiles and the total spread of the values (excluding outliers) as line, box and whiskers, respectively. The version of this figure on the *F1000Research* website is interactive; readers can define the type of whiskers displayed as either Tukey whiskers (1.5 x IQR from 1 ^st^/3 ^rd^ quartile; **A**) or the 10 ^th^–90 ^th^ percentiles ( **B**). The text color code used for the genotypes is analogous to that used in Figure 2. The red horizontal line corresponds to the median value for random walks: 44°. Sample size is 11–12 for each boxplot. No statistical analysis was performed.

Strikingly, the stripe fixation behavior covered the full range from strong fixation (10° average deviation from the stripe) to almost no fixation at all (30°: a random walk generates a 44° score, ^7^) ( Figure 3). We did not perform any statistical tests on this data, as they are already included in the PCA.

**Figure 4.**
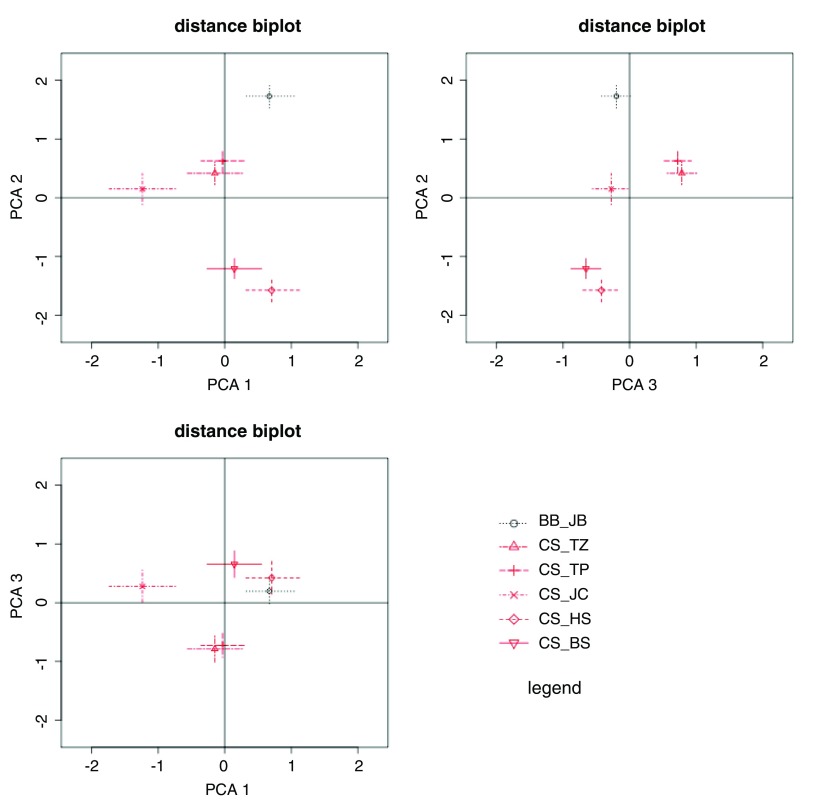
Updating principal component analysis of Canton S strains. Results from the PCA obtained using the same analysis as for Figure 2, but with data uploaded from different laboratories. The version of this figure on the *F1000Research* site is ‘living’; it will automatically re-plot as and when new data for other Canton S strains are submitted, and users can visualize previous versions of this figure. The conclusions of this article only relate to the data available at the time of publication. The prefixes in the key are the initials of the data contributor (except CS_ strains, which were tested by Julien Colomb); full names and affiliations can be found in the figure legend of the article on the *F1000Research* site. The suffixes denote the initials of the principal investigators from where each sub-strain was sourced. The BB_JB (Jose Botella) strain was ordered from the Bloomington stock center (stock #1) approx. seven years ago. BB_JB falls within the range of variability seen so far, but does not appear to clearly group with any of the previously measured strains. **Instructions for adding data:** Click the ‘Submit New Data’ button to the left of the figure on the online version of article and fill in the fields on the form (user registration may be required). 'Uploader Name' should be “First name, Last name”; 'Uploader Lab Address' should be “Department, University, Country”. For 'Genotype', use the initials of the principal investigator of the lab from where the strain originated (this will typically be the initials of the uploader). The data should be uploaded as one metadata and one data file for each fly (click links for template examples). Only one CS strain per lab should be uploaded. Please email BB for additional details on contributing data to this figure.

In order to estimate the variability range of the CS behavior on a larger scale, we have set up a trajectory database to receive data from CS flies in different laboratories, using similar machines and protocols. In Figure 4, we have visualized the result of a PCA over both our and submitted data. Additional data will constantly be added to the analysis after the publication of this article; the interactivity of this figure will allow readers to visualize the data at different points in time.

## Discussion

By analyzing the trajectories of five nominally identical CS sub-strains of *Drosophila melanogaster* in Buridan’s paradigm, we were able to distinguish three different groups of sub-strains. In principle, the differences between the strains could be explained by genetic, epigenetic or environmental differences, or a combination of these factors. All strains were treated similarly in the same laboratory conditions for many generations (4 to 6 years) before being tested. There was no difference in rearing or experimental conditions between the different groups of flies. Taking into account these circumstances, it is a straightforward assumption that the differences in behavior we report here are either genetic or epigenetic in origin. Taking all the measured parameters into account, sub-strain differences were comparable in the two replicates conducted one year apart, even though one of the Principal Components showed a statistically significant (but numerically small) replicate effect. In fact, a separate replication in a different location (Regensburg instead of Berlin), new hardware and a different experimenter (Brembs instead of Colomb, manuscript in preparation, see data and project progress at https://github.com/brembslab/cs_buri), suggests that the spatial parameters, in particular, are relatively constant between replicates, while the temporal activity parameters may vary to some degree. We take these observations as evidence that the differences between the sub-strains are stable over at least several years.

The time elapsed between replicates also seems to suggest that epigenetic changes are rather unlikely. We thus tentatively conclude that the differences between the strains are genetic in origin and have hence begun to sequence the genomes of these five Canton S sub-strains, with marked alterations in all of them (manuscript in preparation, see data and project progress at https://github.com/brembslab/cs_buri). However, epigenetic modification, as well as selection may play a much larger role when studying mutant or transgenic lines which have been outbred to, e.g. a Canton S genetic background. In these cases there may be more or less strong evolutionary forces driving genomic changes.

From the twelve parameters of walking that we tested, stripe deviation showed the most striking variability. Stripe fixation likely depends on multiple parameters, such as the fly’s light/dark preference, their anxiety state, visual acuity, leg motor coordination or effects of wing clipping. It was used as a determining behavioral feature of Buridan’s paradigm ^10^. Our results call for special care with the genetic background of the tested strains when analyzing this behavioral feature.

The numerically small but statistically significant difference between the two replicates (see raw data for individual variables) may be attributed to the differences in test setups and conditions. Since the behavior of the flies did not tend to converge (at least not over the one year time-frame we covered), the different strains apparently did not evolve particular traits to cope with our particular laboratory conditions. It is therefore plausible that such micro-evolution played little role in differentiating the sub-strains in the first place; the major cause for the difference between sub-strains might therefore be founder effects produced when a new fly stock is established, or population bottlenecks in the history of each strain. This hypothesis is also supported by the fact that common descent fails to explain the grouping we found in the PCA. In particular, two strains originating from the Paris lab (CS_TP and CS_JC) showed strikingly different locomotor behavior. This suggests that founder effects or bottlenecks were leading to dramatic alterations of behavior in Buridan’s paradigm. These results raise the question of which other phenotypes might be affected in the numerous CS sub-strains present in laboratories throughout the world.

The results also raise the question, if every laboratory sub-strain is effectively different from any other strain, or if there are groups of sub-strains that remain genetically and behaviorally similar. To examine the degree to which different laboratory strains cluster around certain groups, we are soliciting Buridan data from other laboratories with Canton S strains. The results in Figure 4 will be updated, whenever new data is being uploaded, such that the degree of clustering between sub-strains can be observed. Further research will be required to test the hypothesis that most of the variance between sub-strains of wild type flies is due to genetic differences acquired by founder effects.

Interestingly, the use of a control line may lead to inaccurate interpretation of the data. For example, crammer mutant flies were reported to either show ^11^ or not show ^12^ an appetitive short term memory deficit with identical memory retention scores, because the scores of the control “CS” flies were different in the two studies. Our results further emphasize the need for a more systematic scheme addressing control populations. Existing genetic background differences may indeed explain discrepancies between results obtained in different laboratories, and that the use of the “CS” as a control strain is not enough to achieve comparability or reproducibility. A homogenization of the genetic backgrounds of ‘standard’ control strains would indeed be required.

Fortunately, our experiments suggest that the primary cause for differences in wild-type strains come from founder effects and not laboratory selection. One possible, but logistically challenging solution might be to have a common source for lines used for out-crossing events (including control lines), kept in massive, randomly interbreeding populations, for each lab to purchase at regular intervals. Large stock centers such as the Bloomington stock center would in principle be the candidate locations to implement such a solution. However, the phenotypes of mutations can vary depending on the genetic background within which the mutation is embedded ^13– 15^). The choice of one or multiple reference wild-type strain(s) is therefore not without implications for the future of the field and should be carefully investigated.

## Data availability

The data referenced by this article are under copyright with the following copyright statement: Copyright: © 2015 Colomb J and Brembs B

Data associated with the article are available under the terms of the Creative Commons Zero "No rights reserved" data waiver (CC0 1.0 Public domain dedication). 

figshare: Buridan raw data: Sub-strains of Drosophila Canton-S differ markedly in their locomotor behaviour, doi: 10.6084/m9.figshare.1014264 ^16^

Additional data can be downloaded from Figure 4.

## Supplementary materials

Supplementary figures showing individual performance of each strain for each measurement.

## References

[1] Stern C Schaeffer EW: On primary attributes of alleles in *Drosophila melanogaster*. *Proc Natl Acad Sci U S A.* 1943;29(11):351–61. 10.1073/pnas.29.11.351 16588625 PMC1078632

[2] Benzer S: Behavioral mutants of *Drosophila* isolated by countercurrent distribution. *Proc Natl Acad Sci U S A.* 1967;58(3):1112–9. 10.1073/pnas.58.3.1112 16578662 PMC335755

[3] Crawley JN Belknap JK Collins A: Behavioral phenotypes of inbred mouse strains: implications and recommendations for molecular studies. *Psychopharmacology (Berl).* 1997;132(2):107–24. 10.1007/s002130050327 9266608

[4] Paylor R Crawley JN: Inbred strain differences in prepulse inhibition of the mouse startle response. *Psychopharmacology (Berl).* 1997;132(2):169–80. 10.1007/s002130050333 9266614

[5] Gems D Riddle DL: Defining wild-type life span in *Caenorhabditis elegans*. *J Gerontol A Biol Sci Med Sci.* 2000;55(5):B215–9. 10.1093/gerona/55.5.B215 10819307

[6] Buelthoff H Gotz KG Herre M: Recurrent inversion of visual orientation in the walking fly *Drosophila melanogaster*.Springer Berlin/Heidelberg. *J Comp Physiol A.* 1982;148(4):471–81. 10.1007/BF00619785

[7] Colomb J Reiter L Blaszkiewicz J: Open source tracking and analysis of adult *Drosophila* locomotion in Buridan’s paradigm with and without visual targets. *PLoS One.* 2012;7(8):e42247. 10.1371/journal.pone.0042247 22912692 PMC3415391

[8] Götz KG: Visual guidance in *Drosophila*. *Basic Life Sci.* 1980;16:391–407. 10.1007/978-1-4684-7968-3_28 6779803

[9] Guo A Liu L Xia SZ: Conditioned visual flight orientation in Drosophila: dependence on age, practice and diet. *Learn Mem.* 1996;3(1):49–59. 10.1101/lm.3.1.49 10456076

[10] Phillips AM Smart R Strauss R: The *Drosophila black* enigma: the molecular and behavioural characterization of the *black* ^1^ mutant allele. *Gene.* 2005;351:131–42. 10.1016/j.gene.2005.03.013 15878647

[11] Colomb J Kaiser L Chabaud MA: Parametric and genetic analysis of *Drosophila* appetitive long-term memory and sugar motivation. *Genes Brain Behav.* 2009;8(4):407–15. 10.1111/j.1601-183X.2009.00482.x 19220480

[12] Krashes MJ Waddell S: Rapid consolidation to a radish and protein synthesis-dependent long-term memory after single-session appetitive olfactory conditioning in *Drosophila*. *J Neurosci.* 2008;28(12):3103–13. 10.1523/JNEUROSCI.5333-07.2008 18354013 PMC2516741

[13] Ivanco TL Greenough WT: Altered mossy fiber distributions in adult *Fmr1* (FVB) knockout mice. *Hippocampus.* 2002;12(1):47–54. 10.1002/hipo.10004 11918288

[14] Mineur YS Sluyter F de Wit S: Behavioral and neuroanatomical characterization of the *Fmr1* knockout mouse. *Hippocampus.* 2002;12(1):39–46. 10.1002/hipo.10005 11918286

[15] Lipp HP Wolfer DP: Genetic background problems in the analysis of cognitive and neuronal changes in genetically modified mice. *Clin Neurosci Res.* 2003;3(4–5):223–31. 10.1016/S1566-2772(03)00096-3

[16] Colomb J Brembs B: Buridan raw data: Sub-strains of *Drosophila* Canton-S differ markedly in their locomotor behavior. *figshare.* 2014 Data Source 10.12688/f1000research.4263.1 PMC4156027 25210619

