# Supplementary material for: Sub-strains of Drosophila Canton-S differ markedly in their locomotor behavior
Source: F1000Res. 2015 Apr 21;3:176. Originally published 2014 Jul 30. [Version 2] doi: 10.12688/f1000research.4263.2 (PMC4156027; doi:10.12688/f1000research.4263.2)
Supplement: Supplementary file 2 [file f1000research-3-6750-s0001.tgz › 9ec9cdb9-bb1c-4d43-bf1d-041ab3f7c408.pdf]

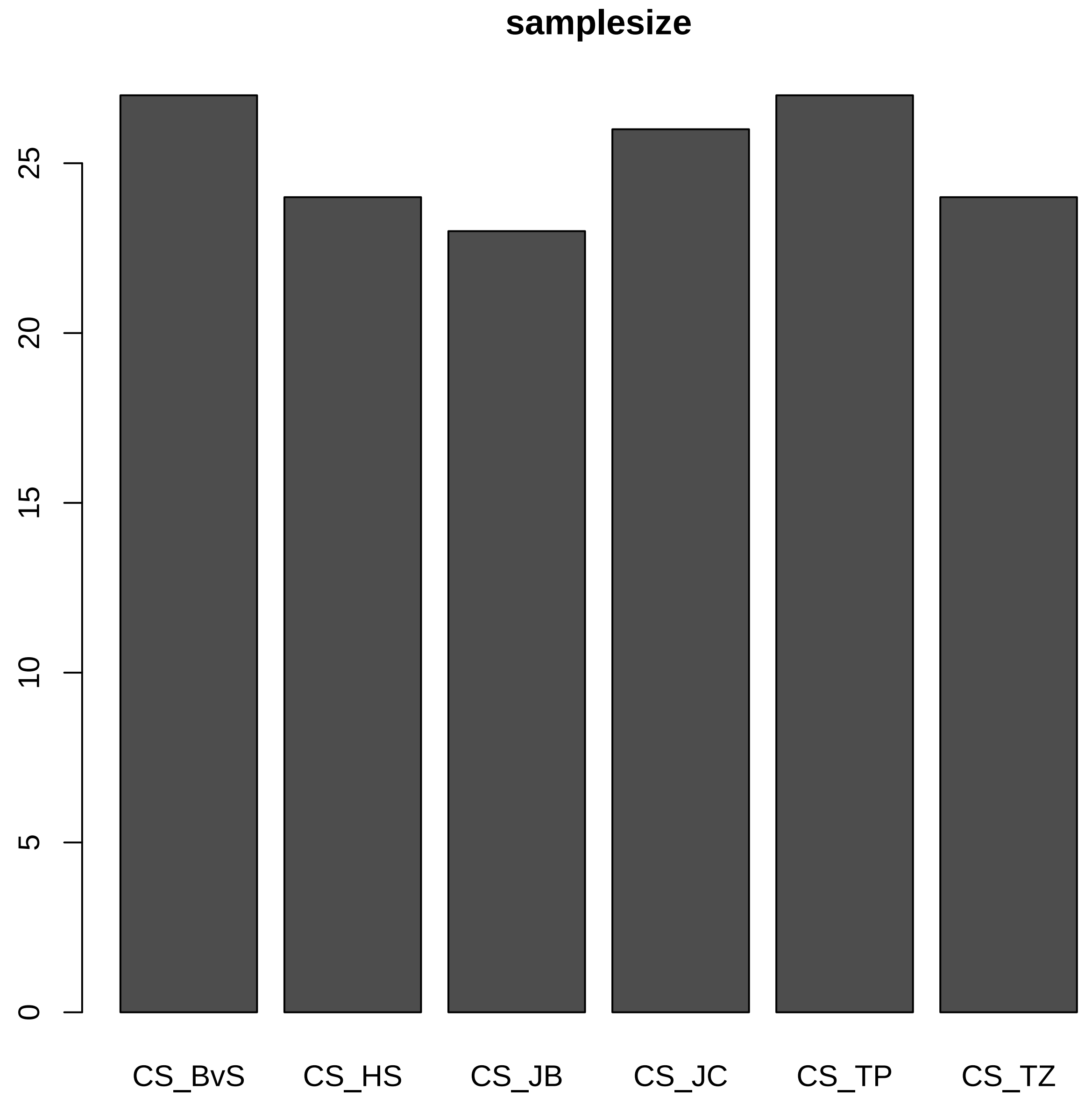

samplesize
0
5
10
15
20
25
CS_BvS
CS_HS
CS_JB
CS_JC
CS_TP
CS_TZ

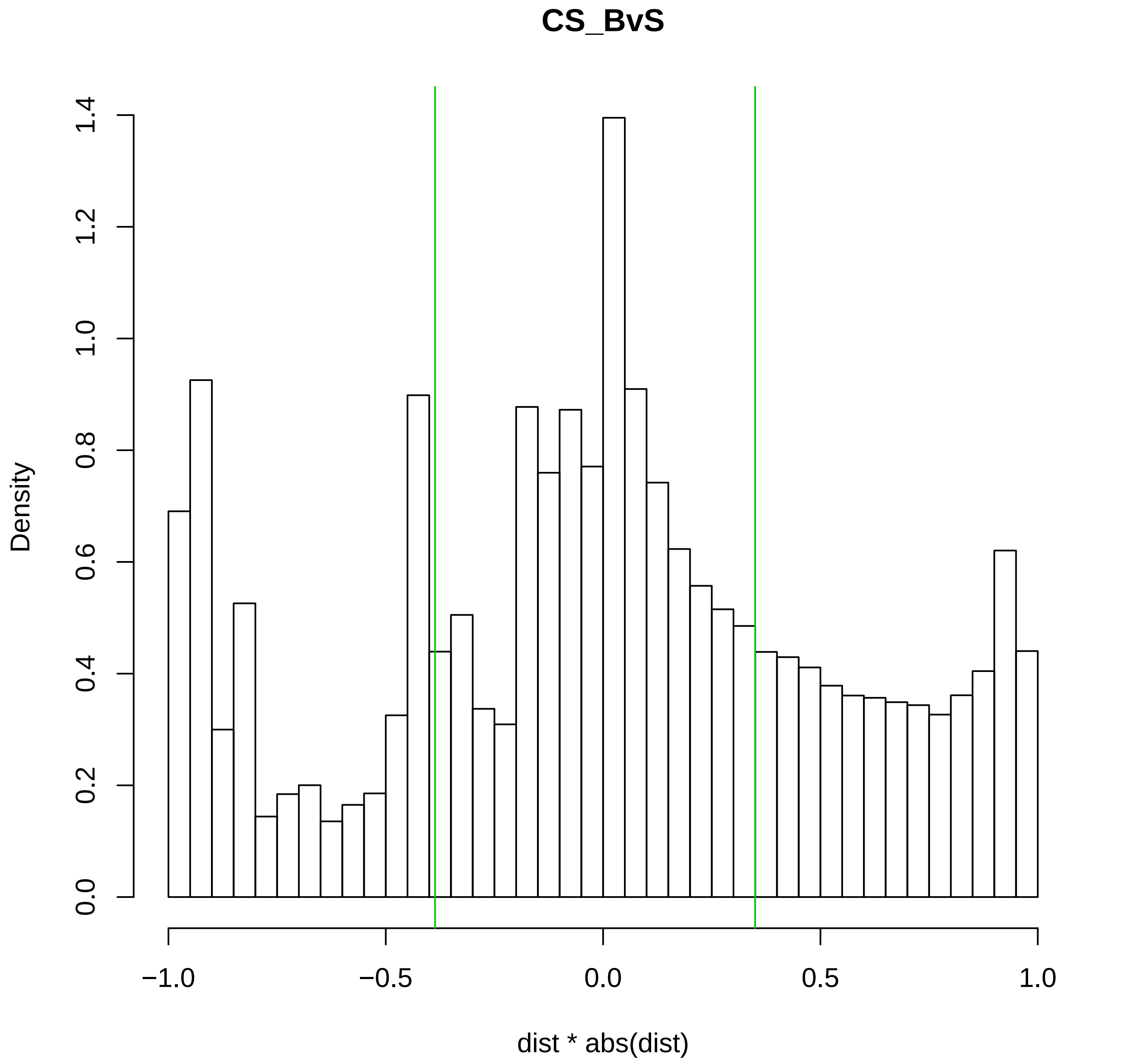
CS_BvS
Density
0.0
0.2
0.4
0.6
0.8
1.0
1.2
1.4
−1.0
−0.5
0.0
0.5
1.0
dist * abs(dist)

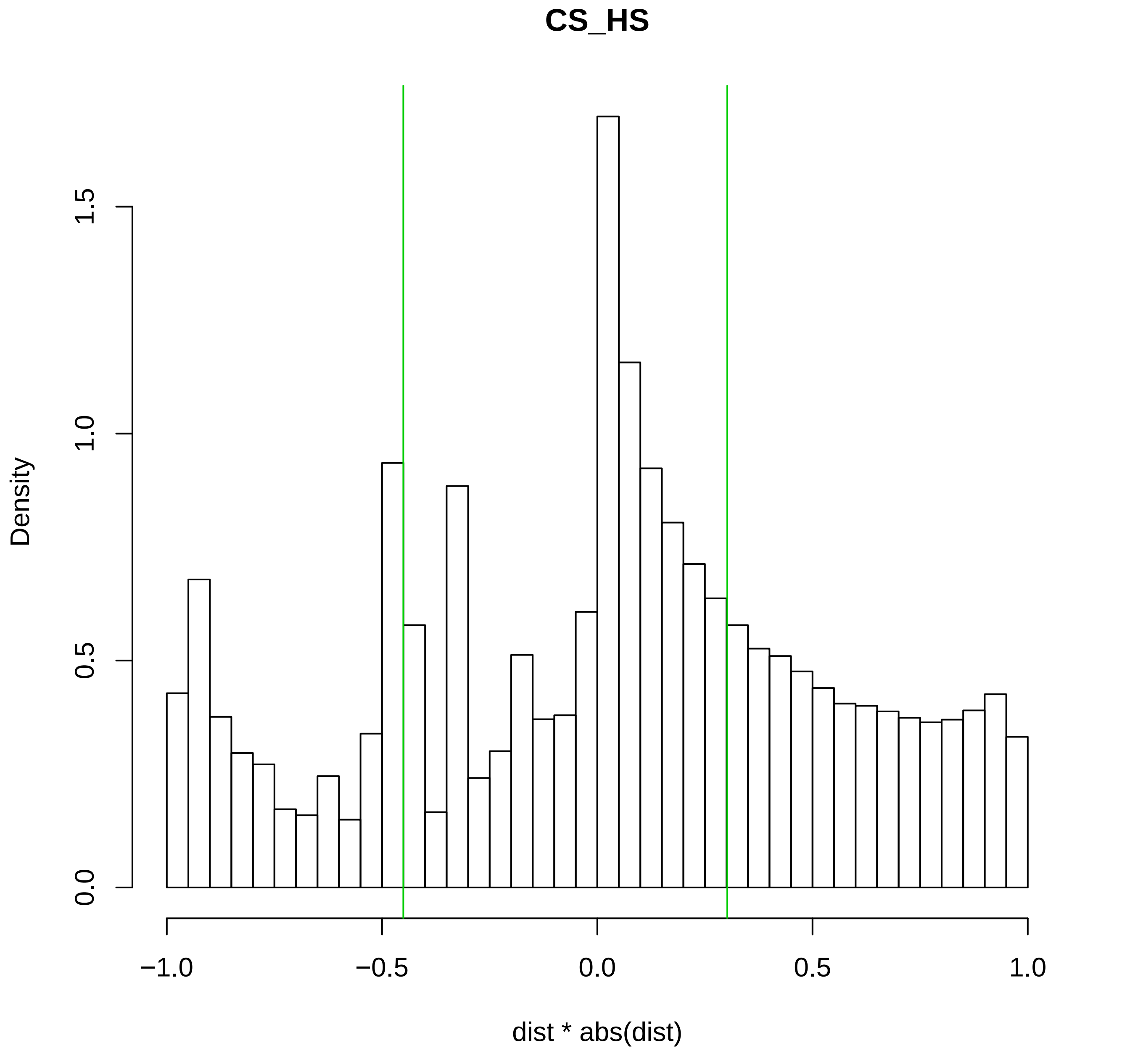
CS_HS
Density
0.0
0.5
1.0
1.5
−1.0
−0.5
0.0
0.5
1.0
dist * abs(dist)

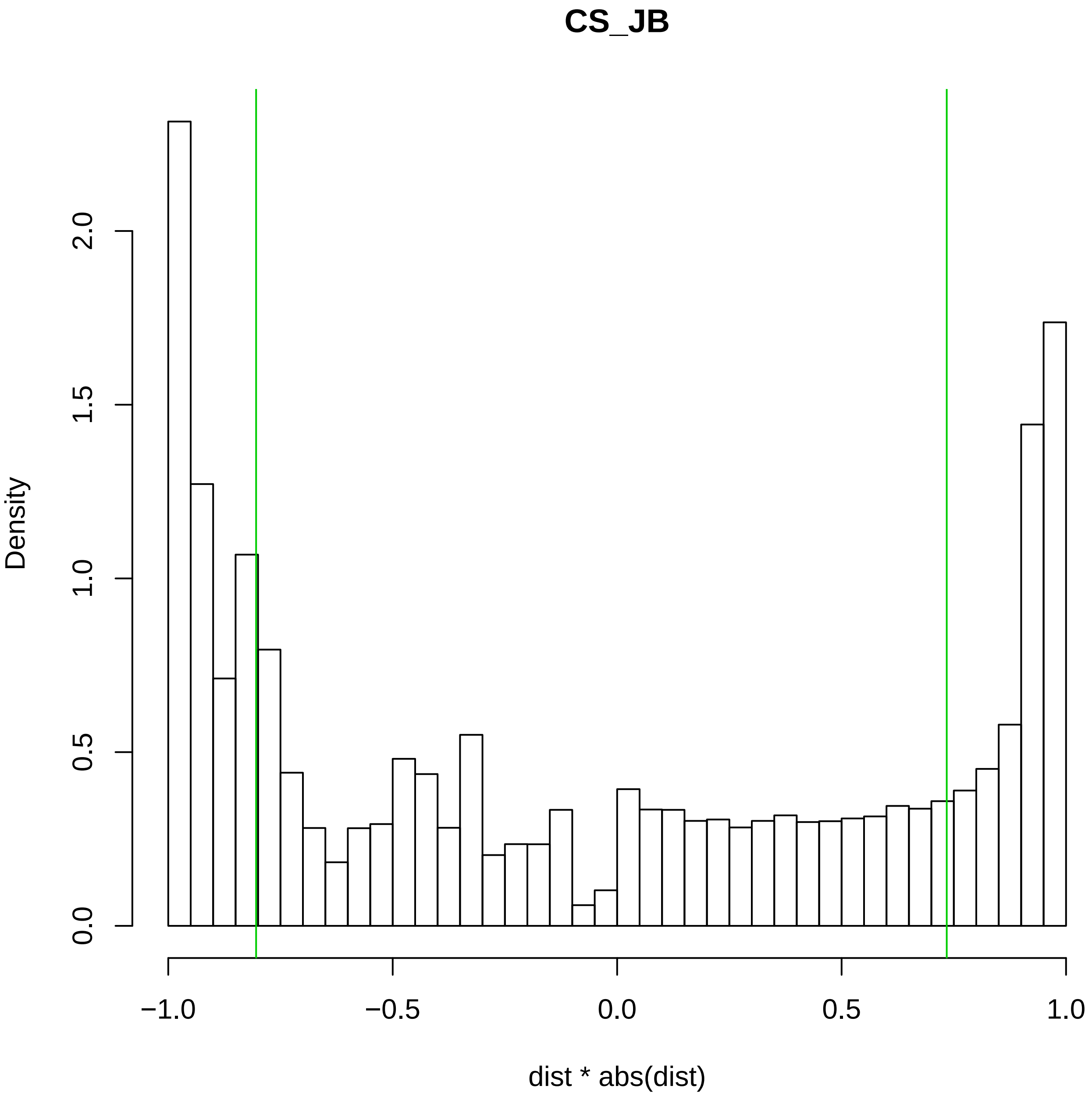
CS_JB
Density
0.0
0.5
1.0
1.5
2.0
−1.0
−0.5
0.0
0.5
1.0
dist * abs(dist)

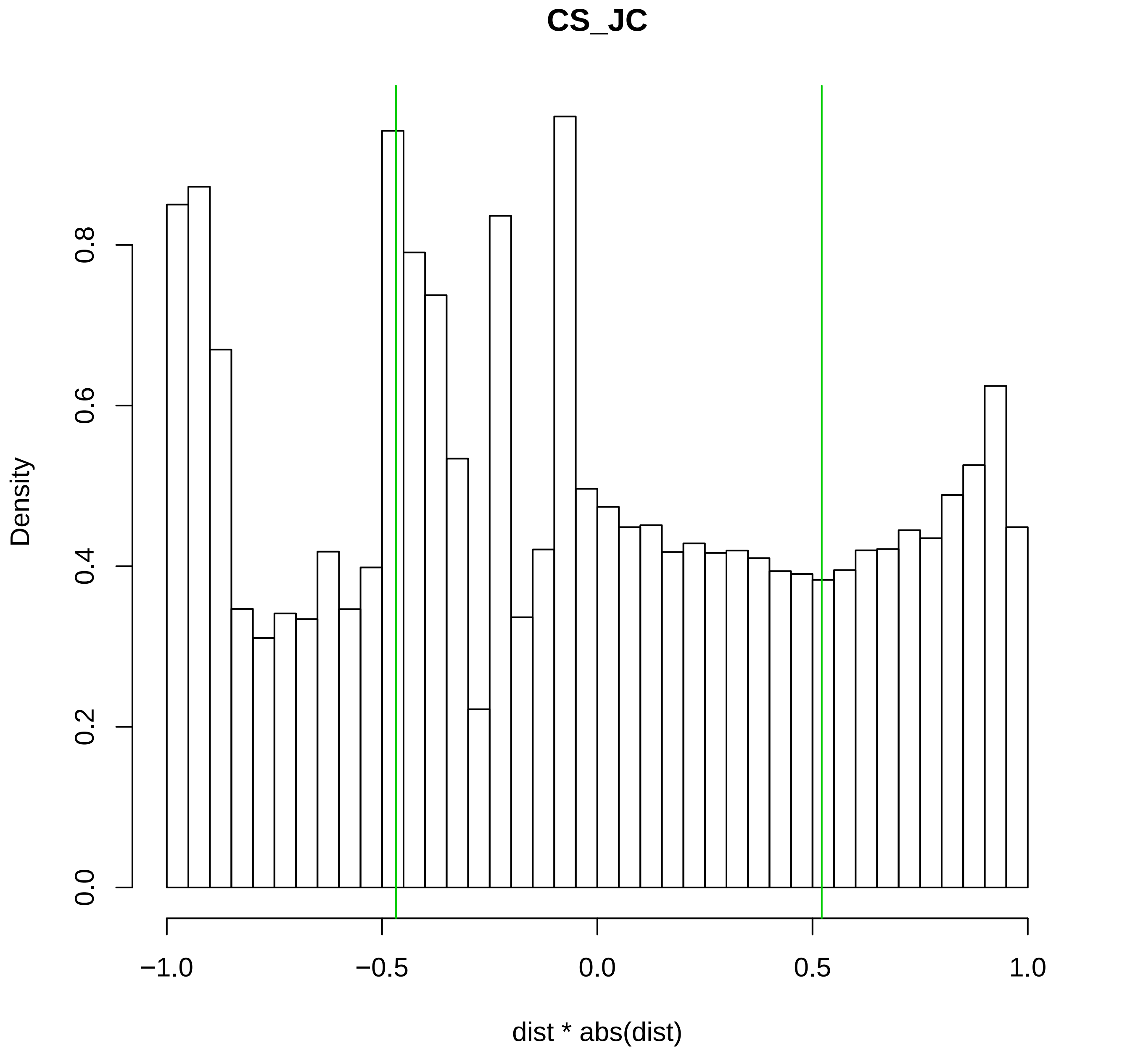
CS_JC
Density
0.0
0.2
0.4
0.6
0.8
−1.0
−0.5
0.0
0.5
1.0
dist * abs(dist)

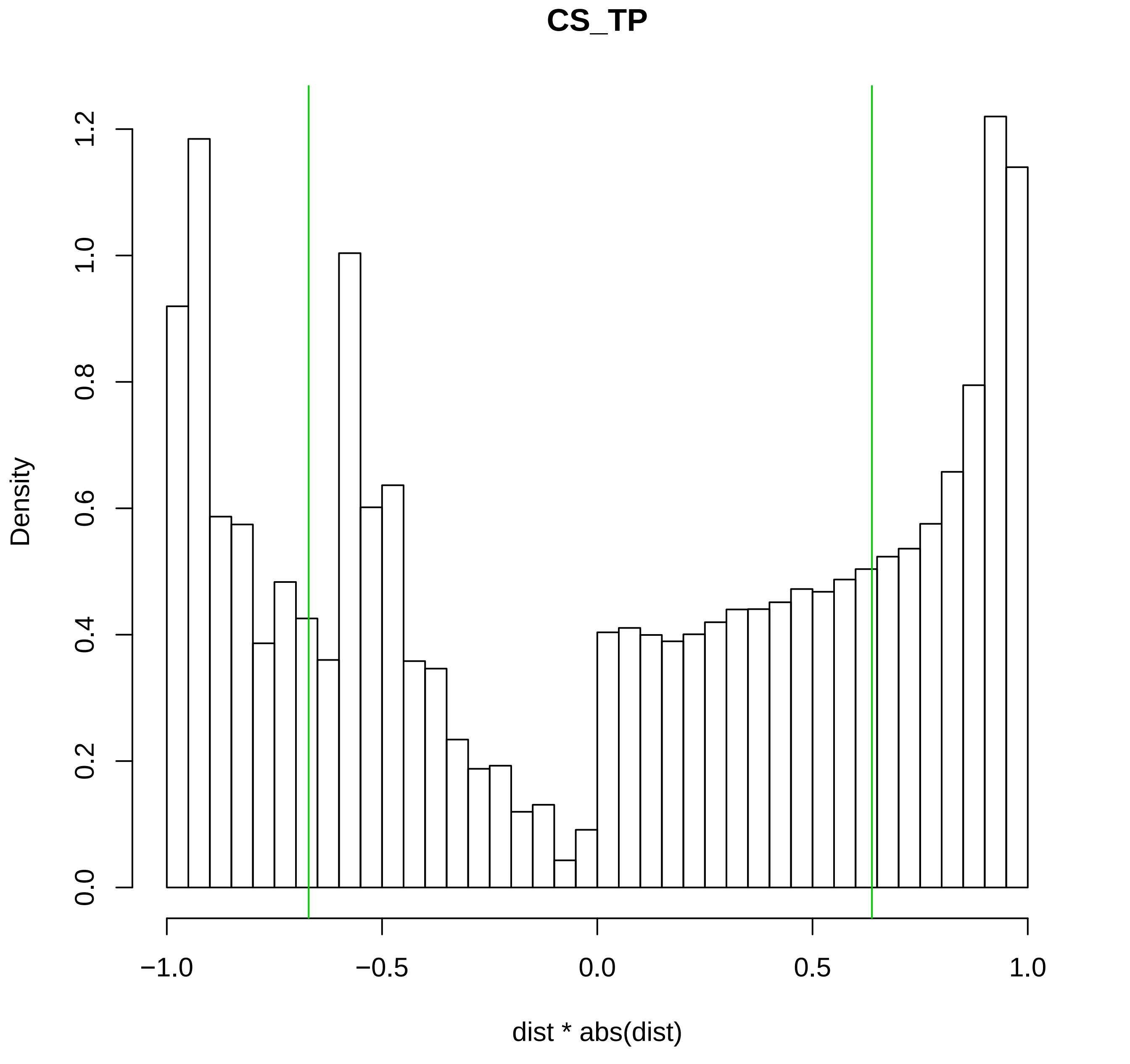

CS_TP
Density
0.0
0.2
0.4
0.6
0.8
1.0
1.2
−1.0
−0.5
0.0
0.5
1.0
dist * abs(dist)

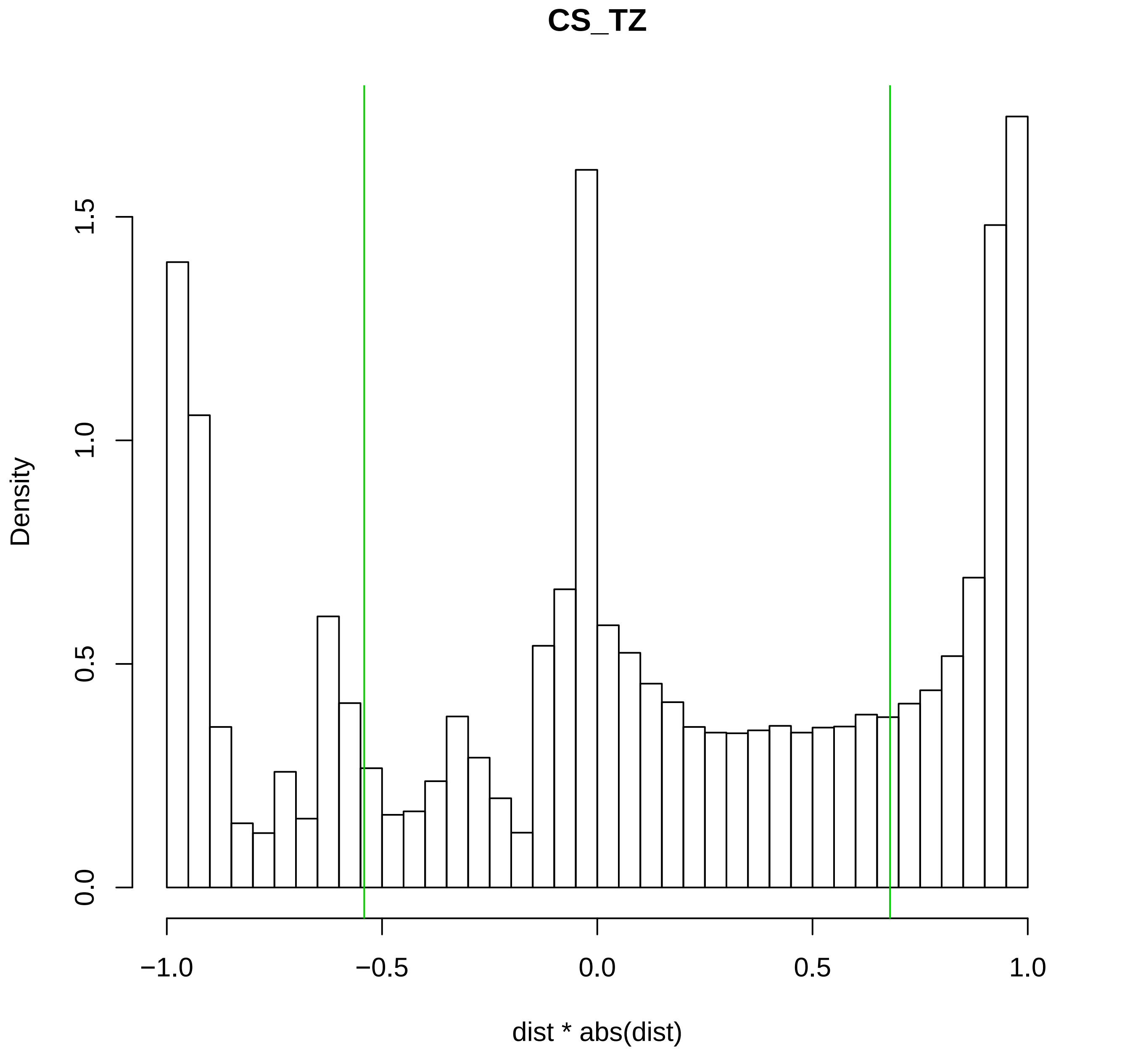

CS_TZ
Density
0.0
0.5
1.0
1.5
−1.0
−0.5
0.0
0.5
1.0
dist * abs(dist)

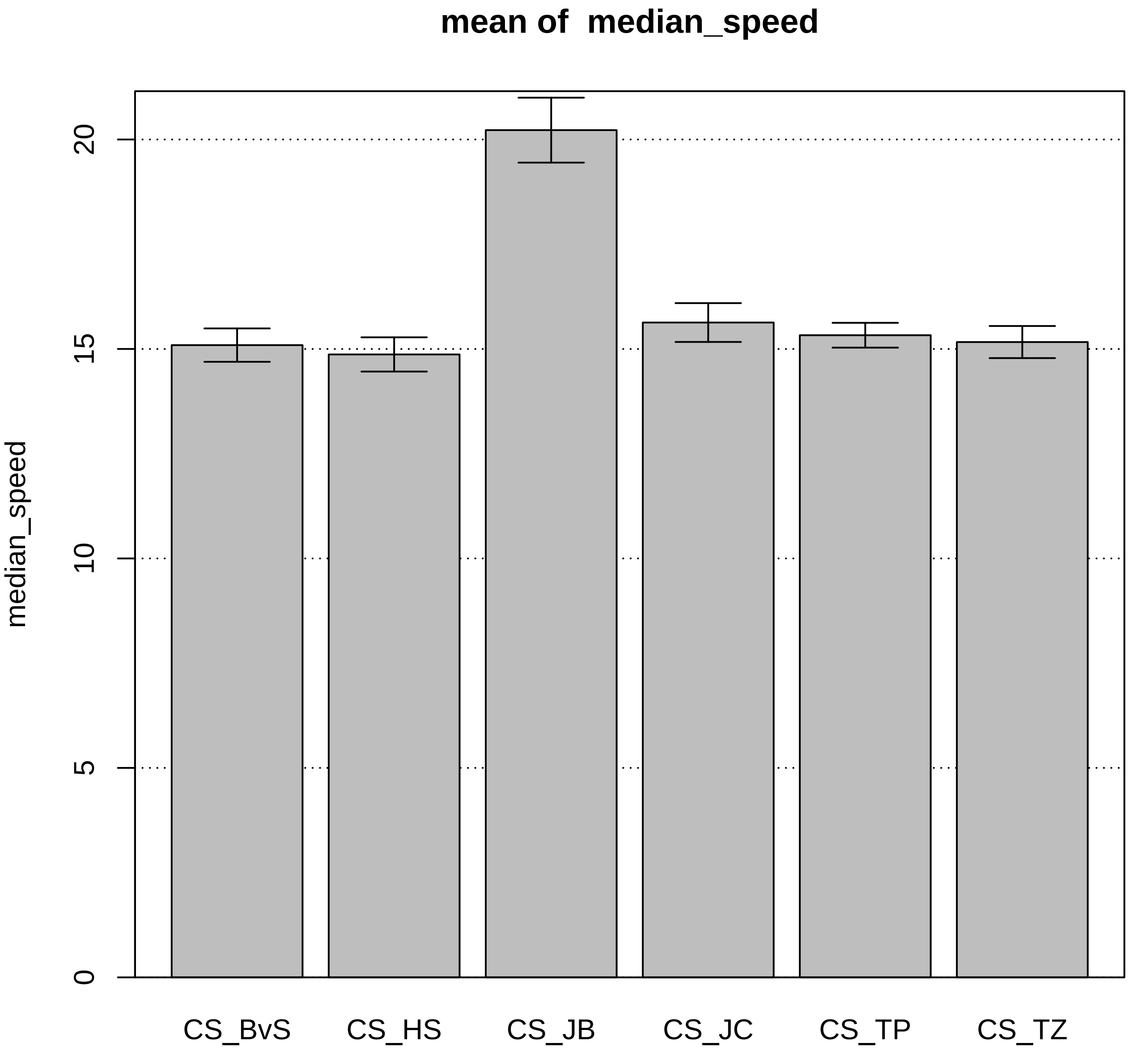
mean of median_speed
median_speed
0
5
10
15
20
CS_BvS
CS_HS
CS_JB
CS_JC
CS_TP
CS_TZ

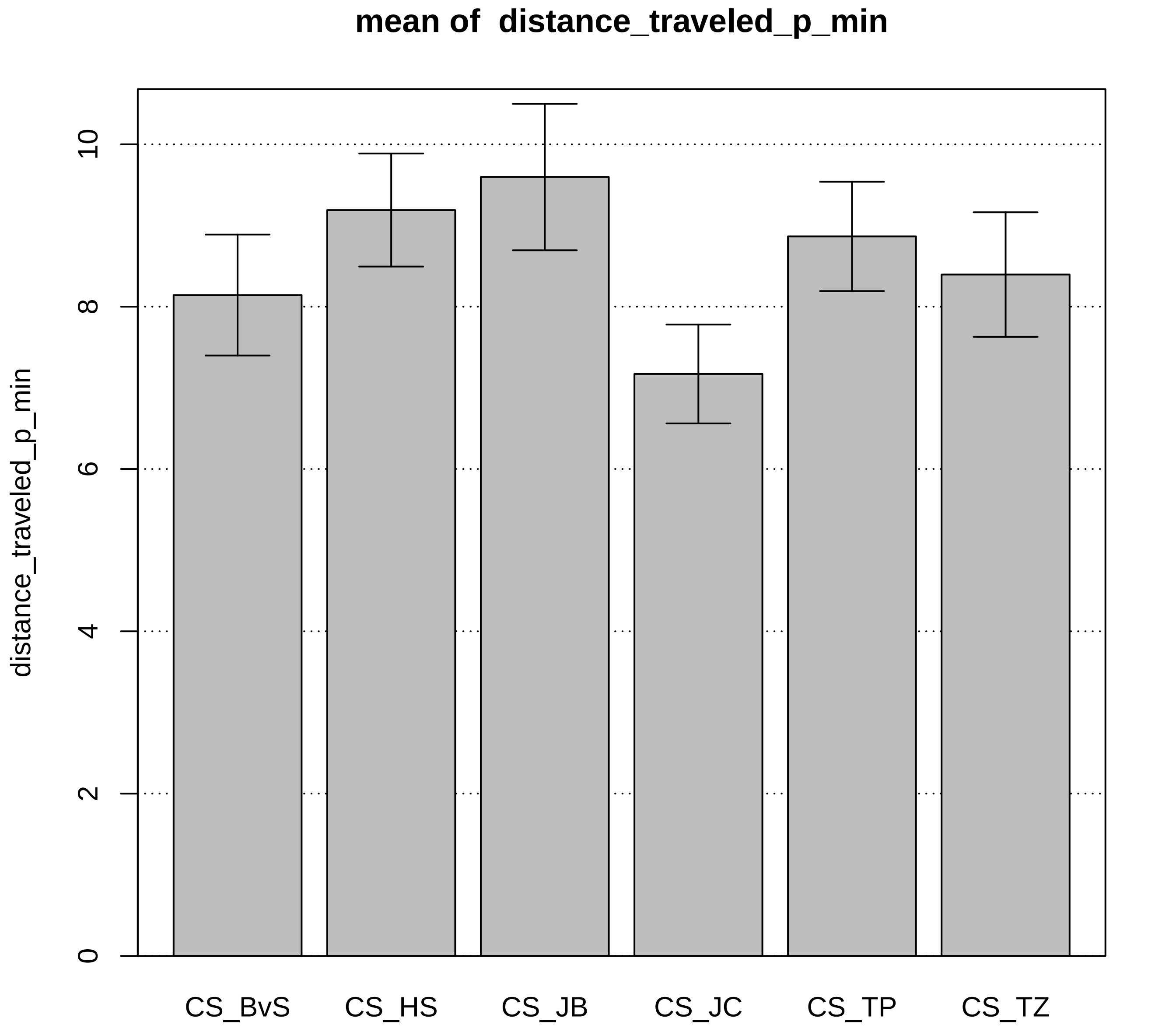
mean of distance_traveled_p_min
distance_traveled_p_min
0
2
4
6
8
10
CS_BvS
CS_HS
CS_JB
CS_JC
CS_TP
CS_TZ

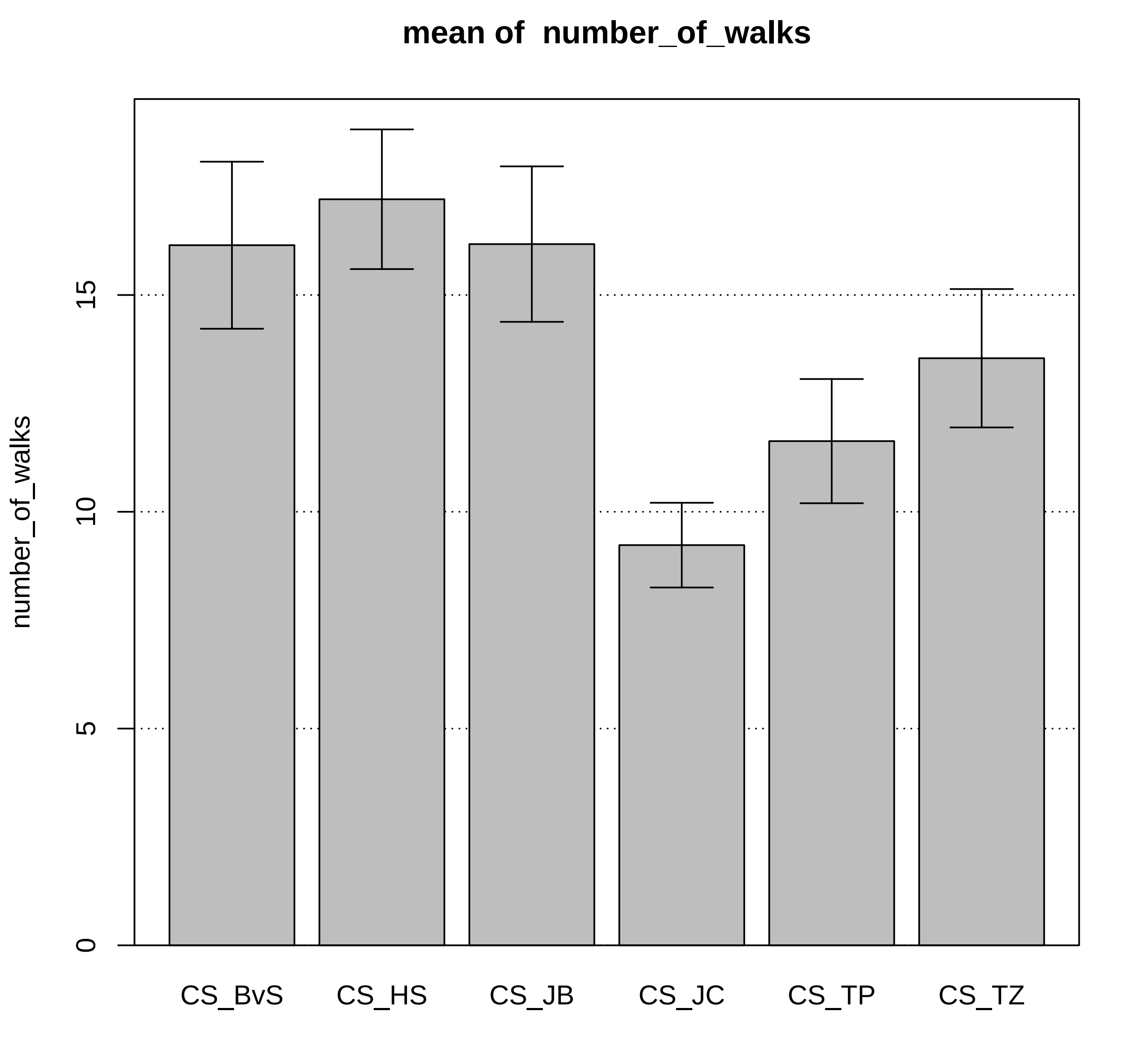
mean of number_of_walks
number_of_walks
0
5
10
15
CS_BvS
CS_HS
CS_JB
CS_JC
CS_TP
CS_TZ

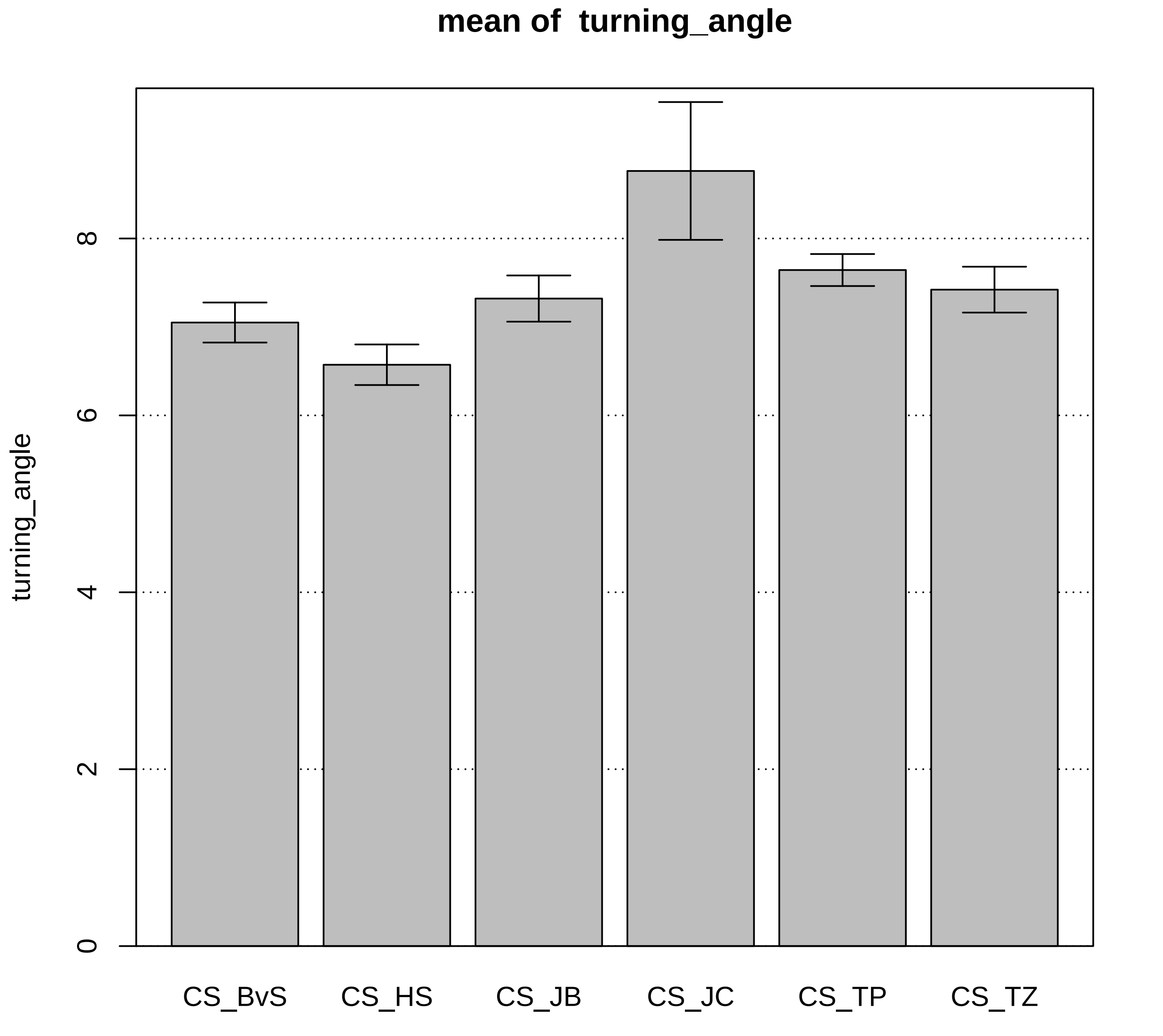
mean of turning_angle
turning_angle
0
2
4
6
8
CS_BvS
CS_HS
CS_JB
CS_JC
CS_TP
CS_TZ

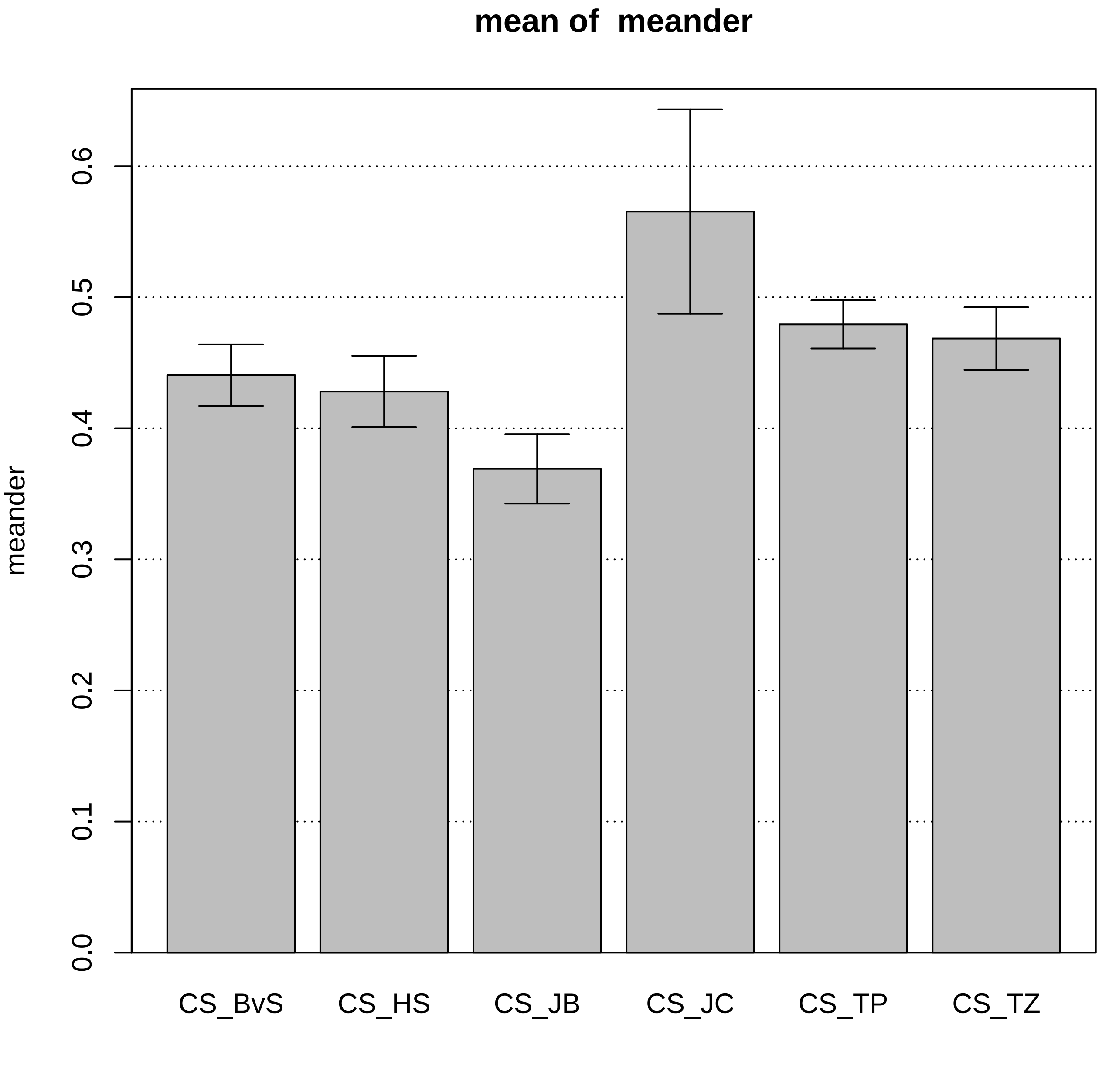
mean of meander
meander
0.0
0.1
0.2
0.3
0.4
0.5
0.6
CS_BvS
CS_HS
CS_JB
CS_JC
CS_TP
CS_TZ

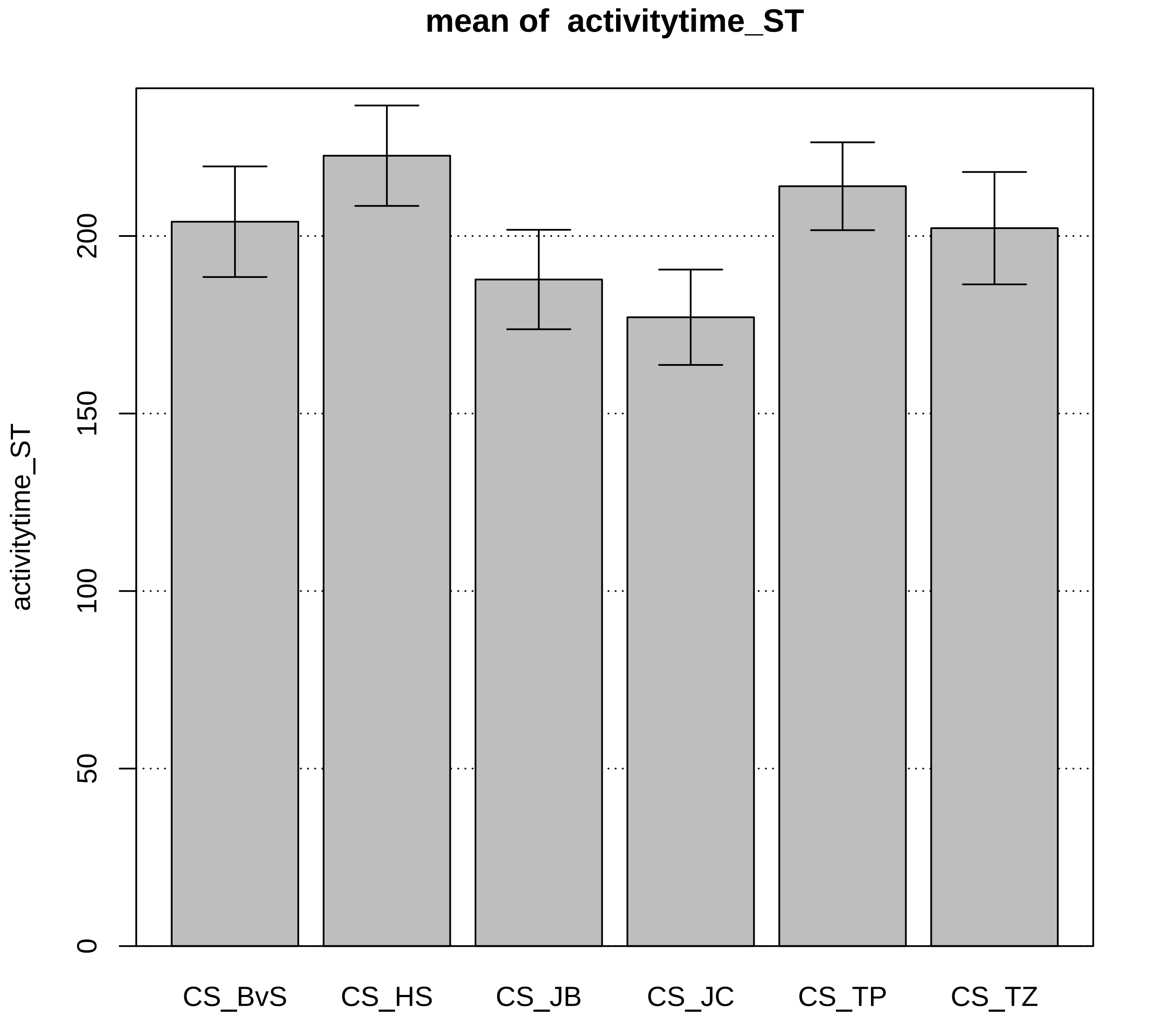
mean of activitytime_ST
activitytime_ST
0
50
100
150
200
CS_BvS
CS_HS
CS_JB
CS_JC
CS_TP
CS_TZ

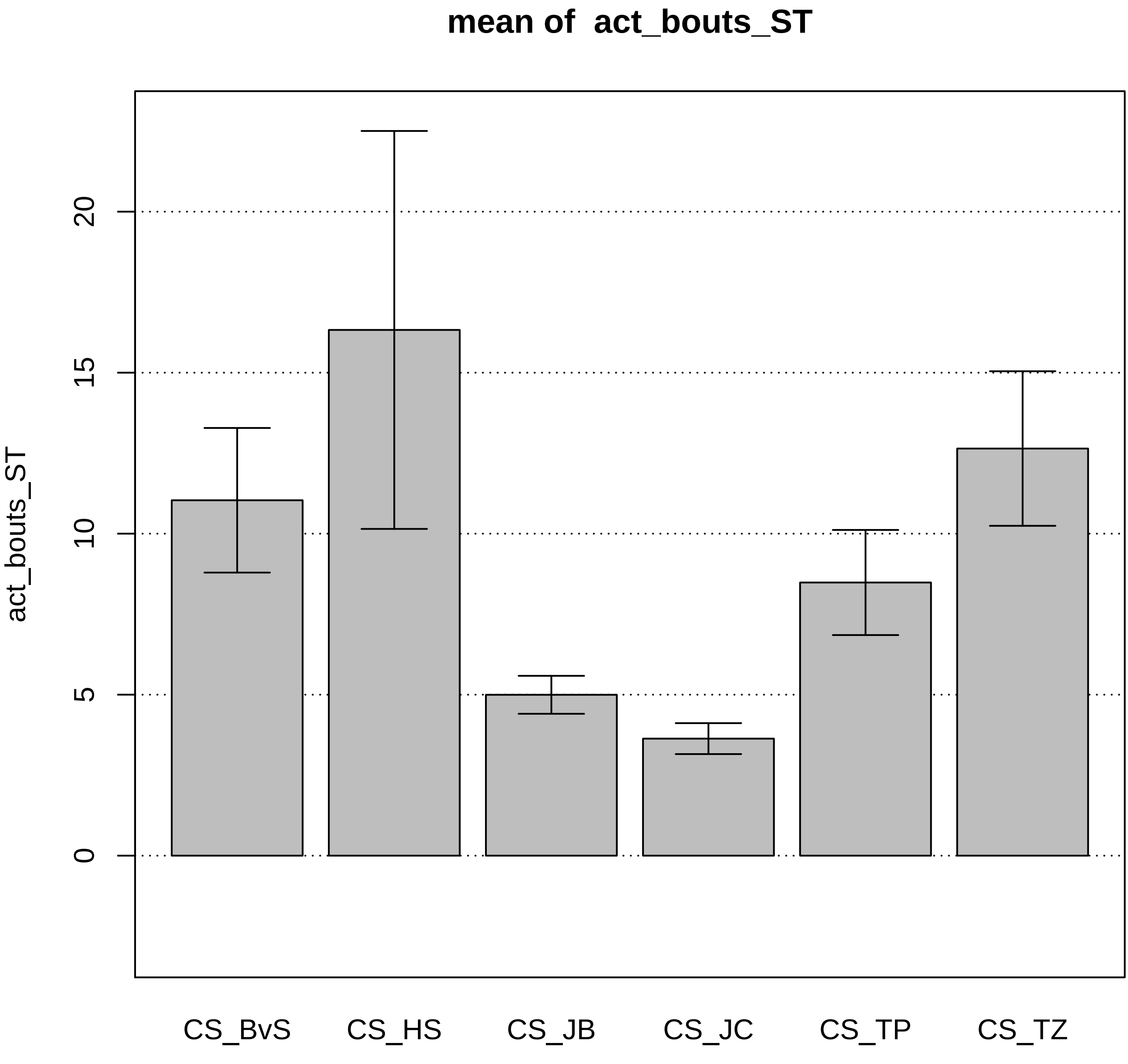

mean of act_bouts_ST
act_bouts_ST
0
5
10
15
20
CS_BvS
CS_HS
CS_JB
CS_JC
CS_TP
CS_TZ

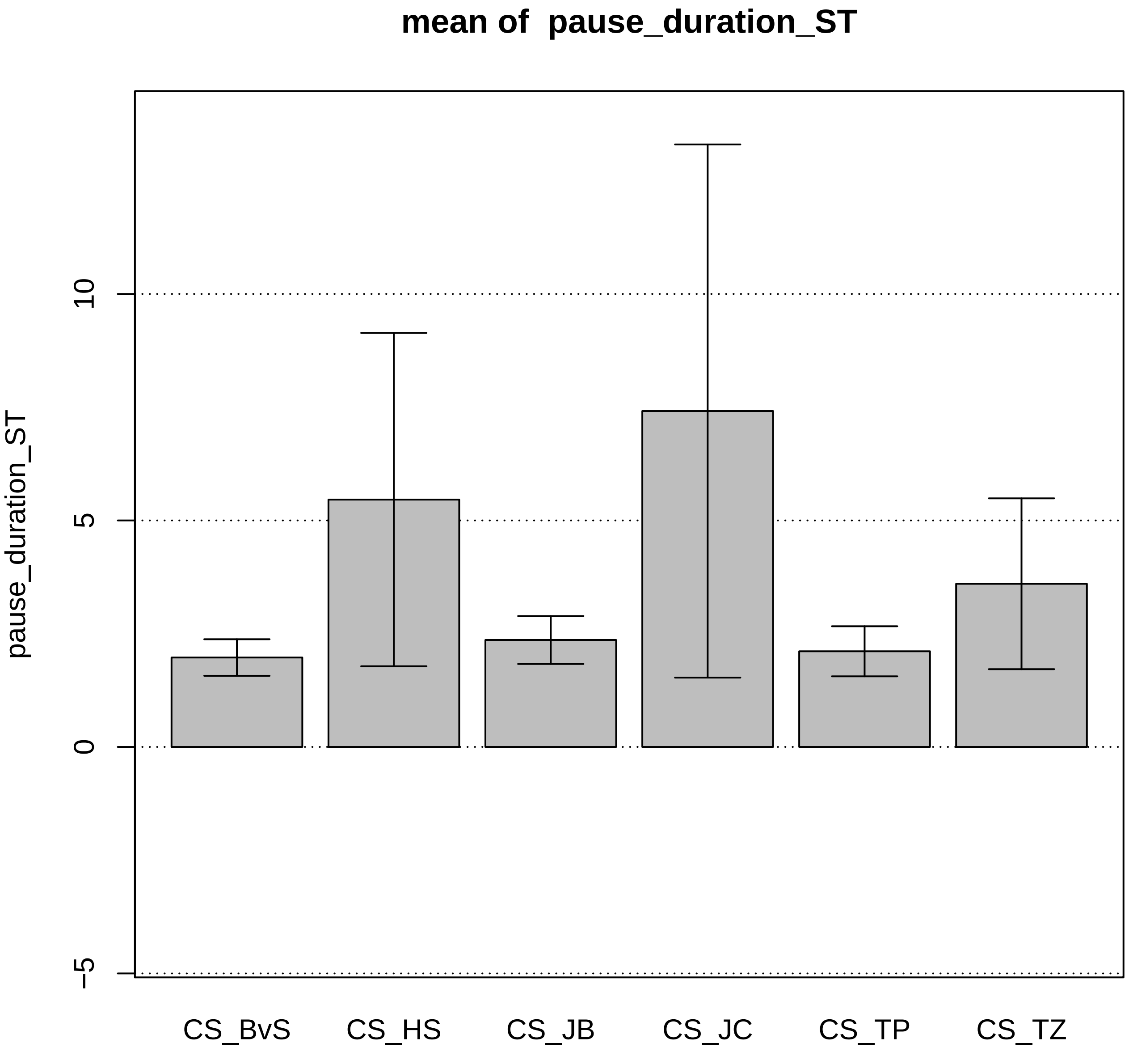

mean of pause_duration_ST
pause_duration_ST
10
5
0
−5
CS_BvS
CS_HS
CS_JB
CS_JC
CS_TP
CS_TZ

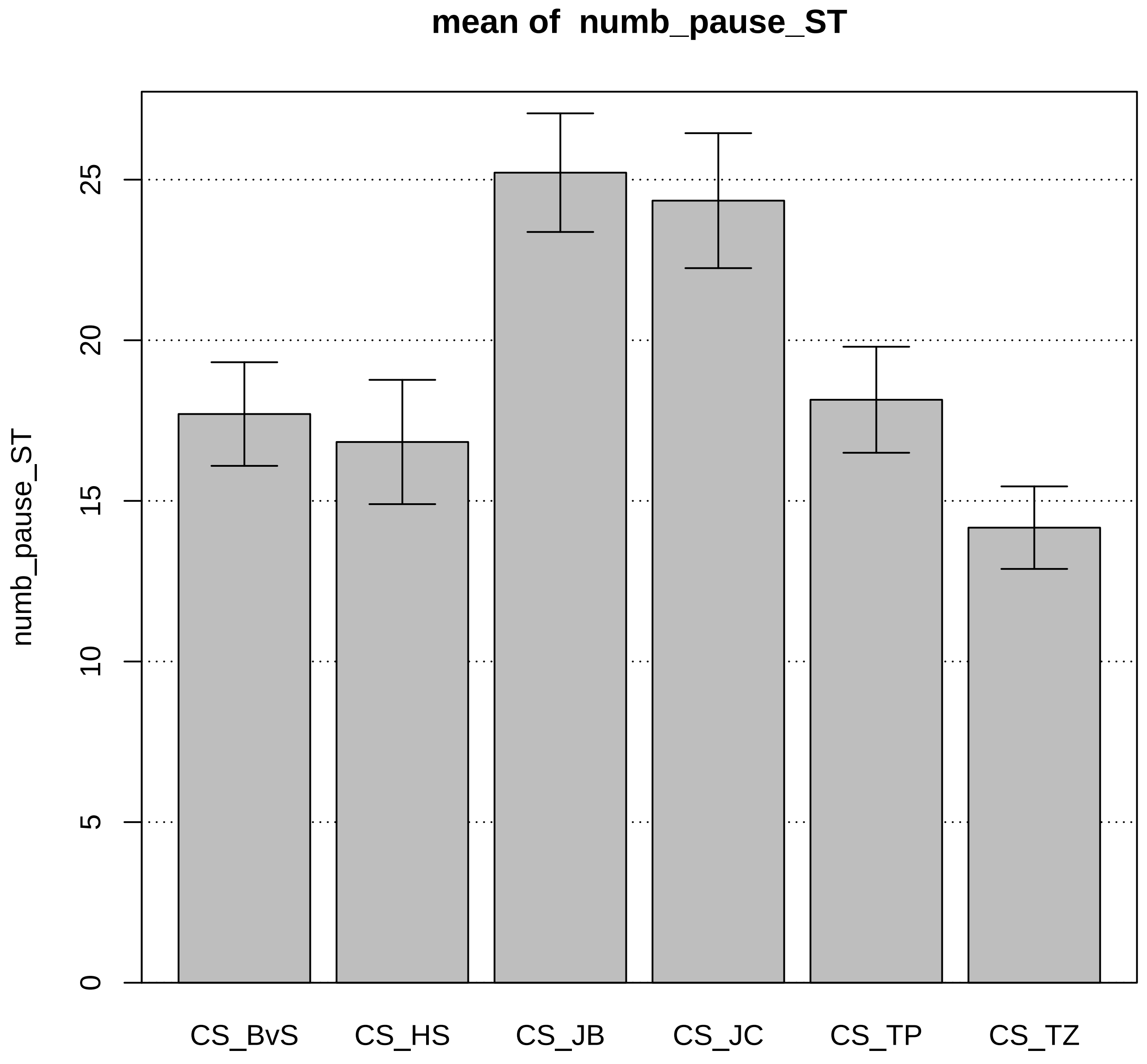
mean of numb_pause_ST
numb_pause_ST
0
5
10
15
20
25
CS_BvS
CS_HS
CS_JB
CS_JC
CS_TP
CS_TZ

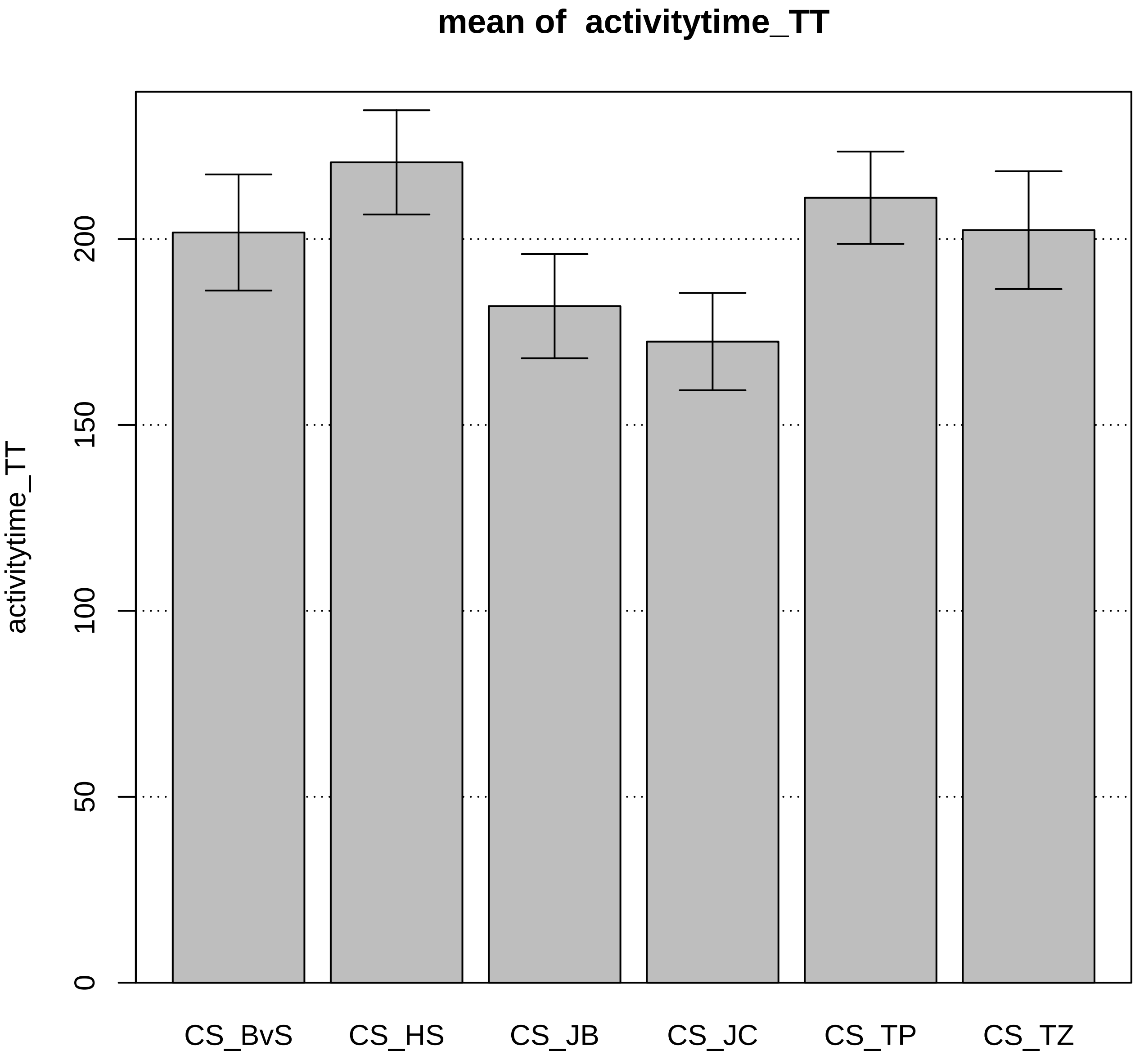
mean of activitytime_TT
activitytime_TT
0
50
100
150
200
CS_BvS
CS_HS
CS_JB
CS_JC
CS_TP
CS_TZ

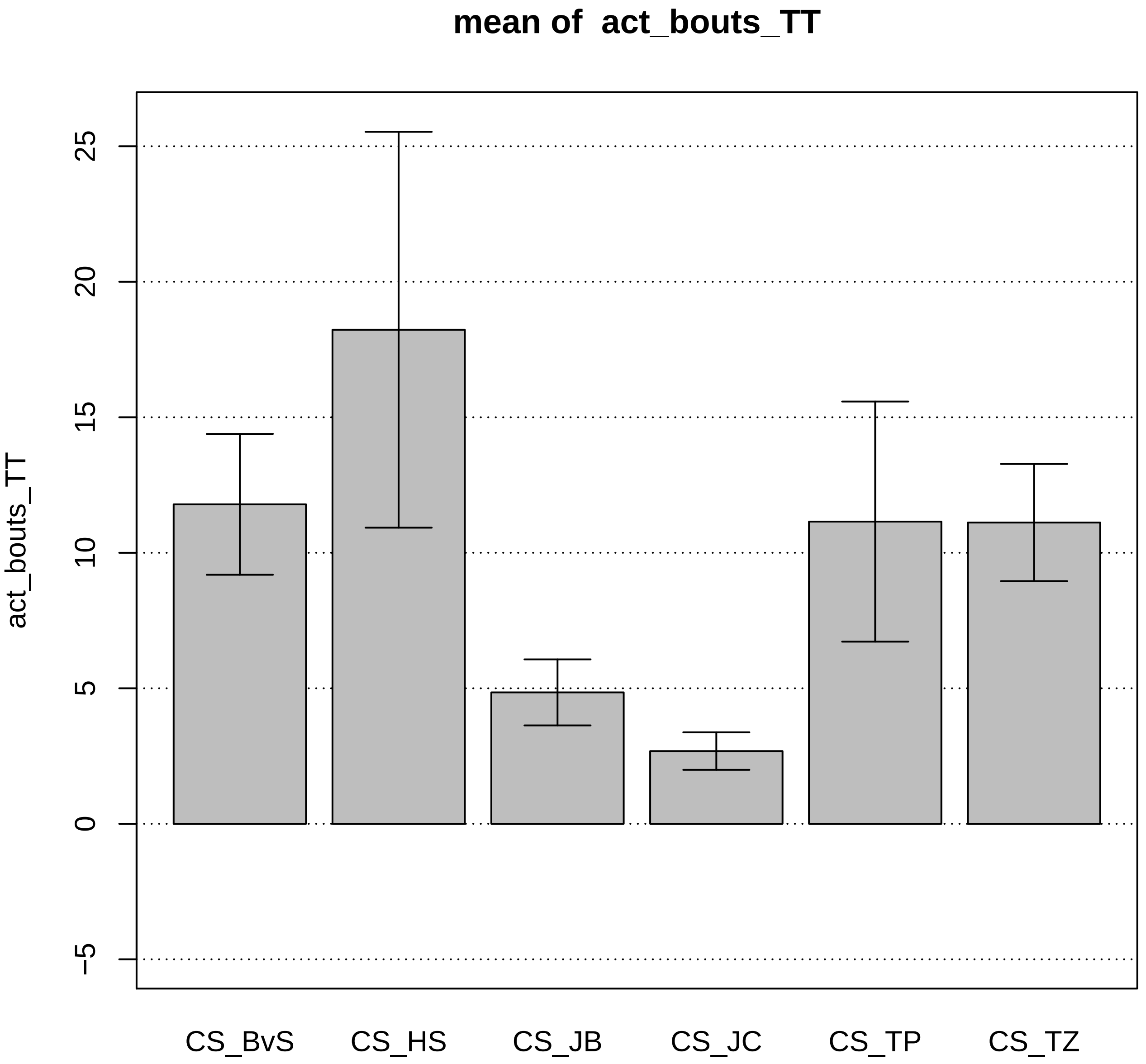
mean of act_bouts_TT
act_bouts_TT
−5
0
5
10
15
20
25
CS_BvS
CS_HS
CS_JB
CS_JC
CS_TP
CS_TZ

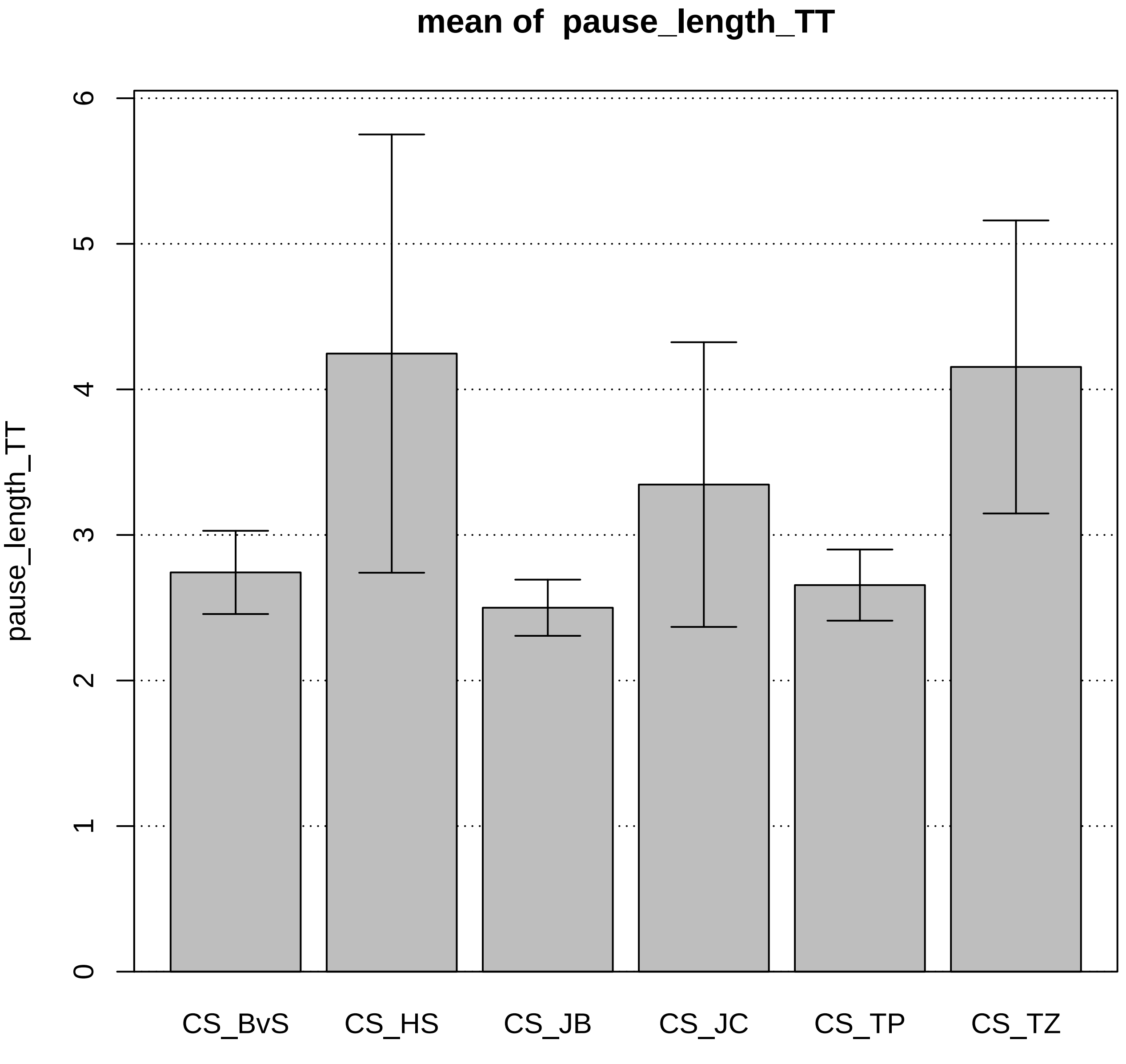
mean of pause_length_TT
pause_length_TT
0
1
2
3
4
5
6
CS_BvS
CS_HS
CS_JB
CS_JC
CS_TP
CS_TZ

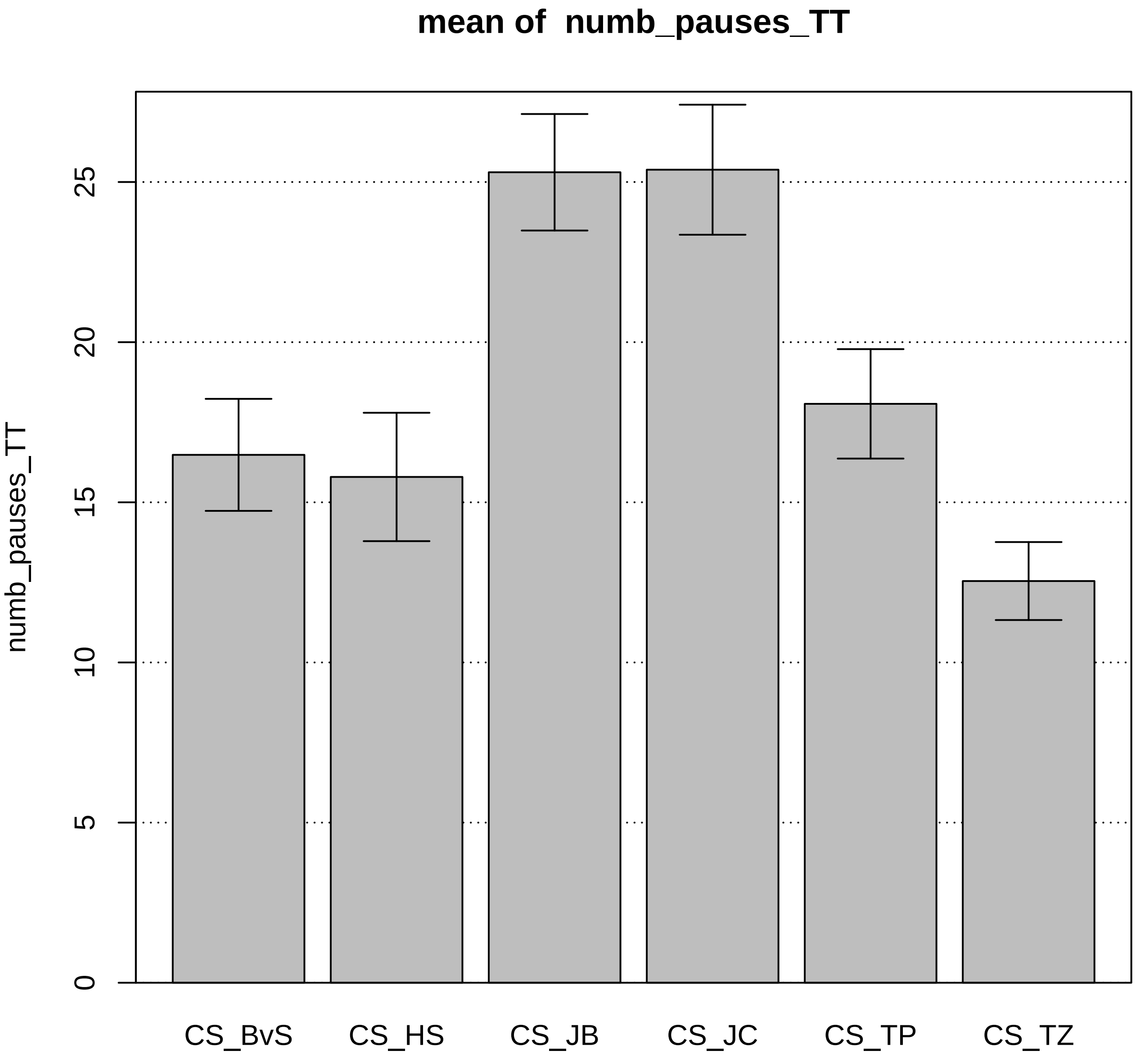
mean of numb_pauses_TT
numb_pauses_TT
0
5
10
15
20
25
CS_BvS
CS_HS
CS_JB
CS_JC
CS_TP
CS_TZ

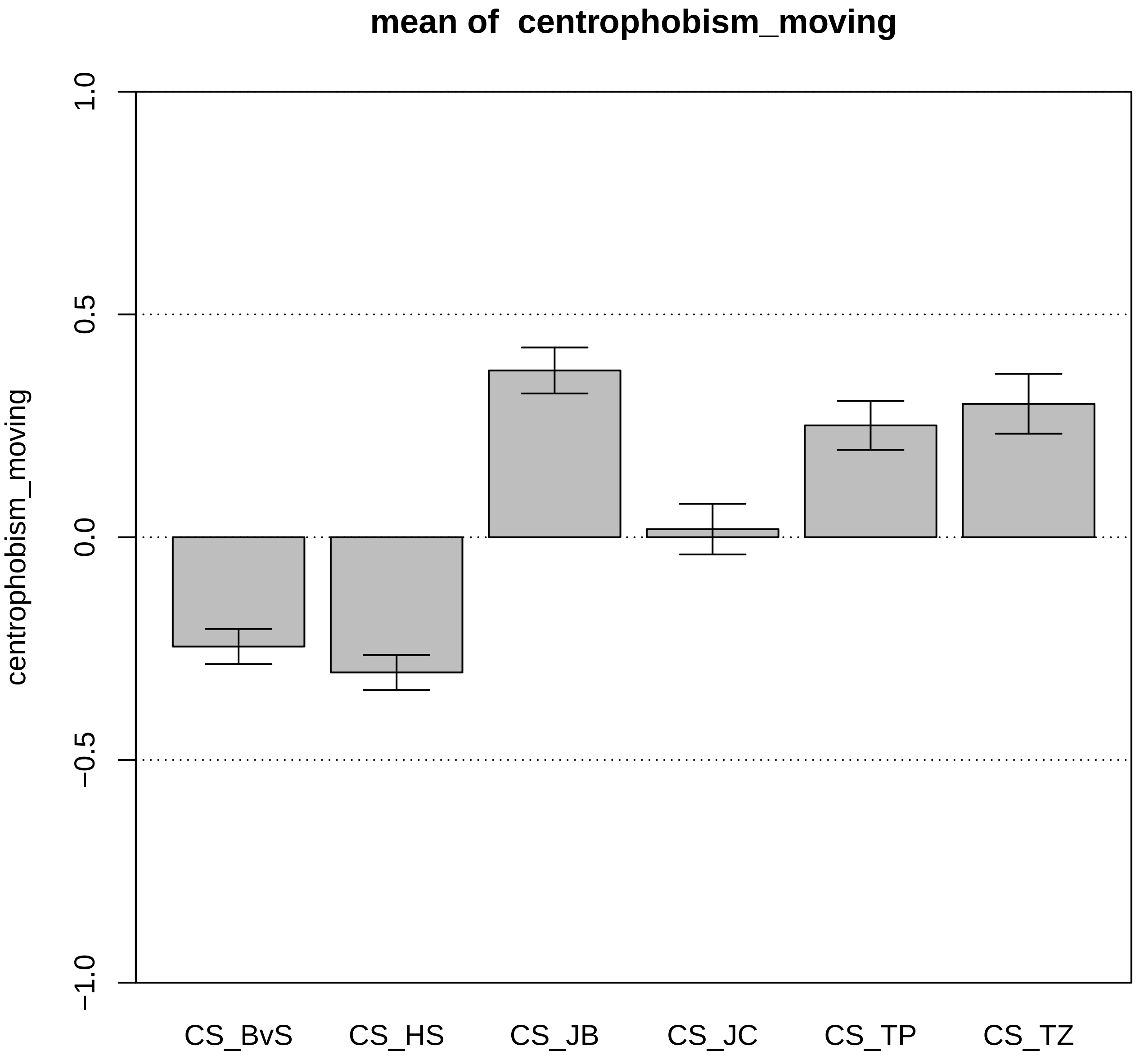
mean of centrophobism_moving
centrophobism_moving
1.0
0.5
0.0
−0.5
−1.0
CS_BvS
CS_HS
CS_JB
CS_JC
CS_TP
CS_TZ

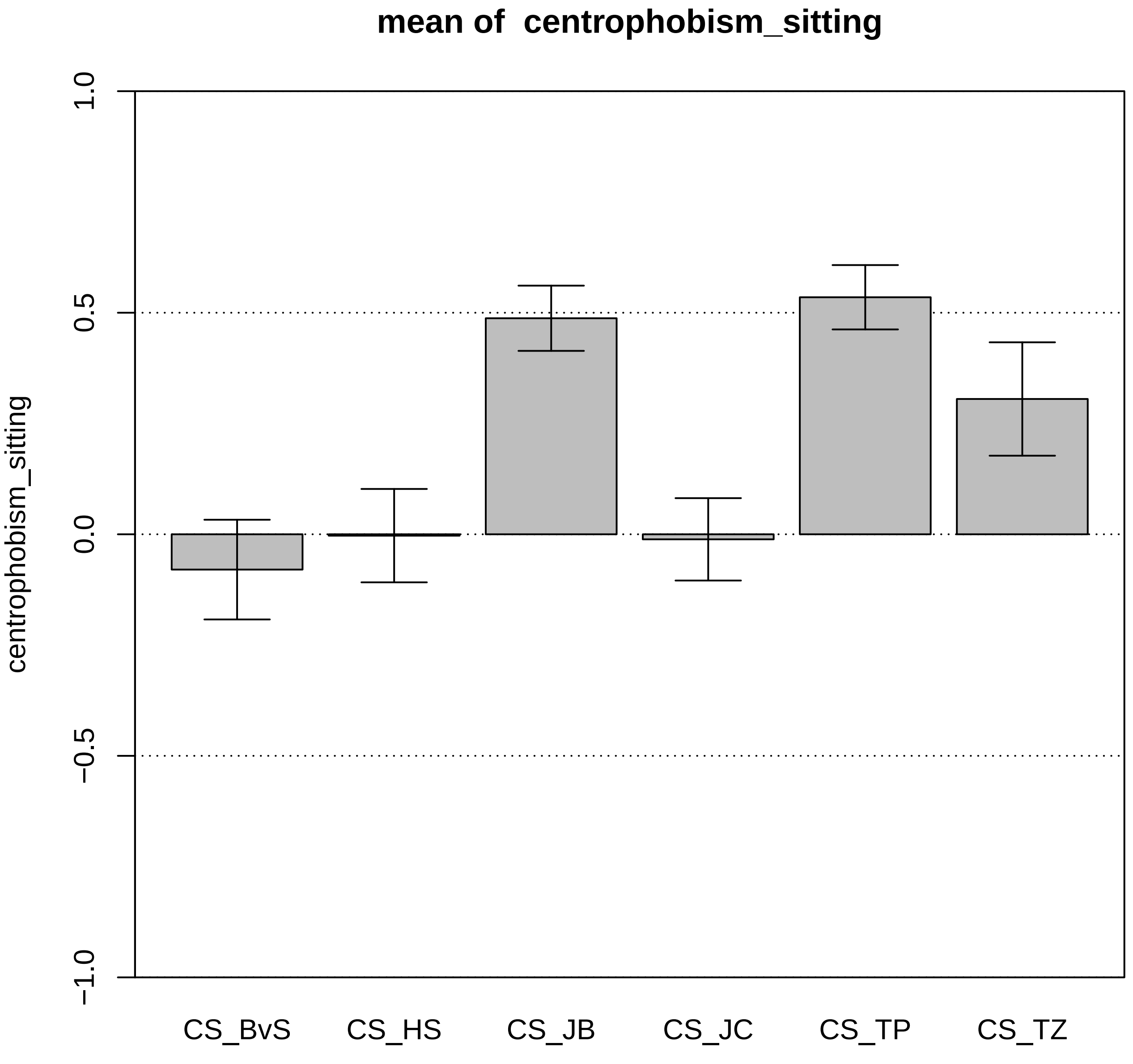

mean of centrophobism_sitting
centrophobism_sitting
1.0
0.5
0.0
−0.5
−1.0
CS_BvS
CS_HS
CS_JB
CS_JC
CS_TP
CS_TZ

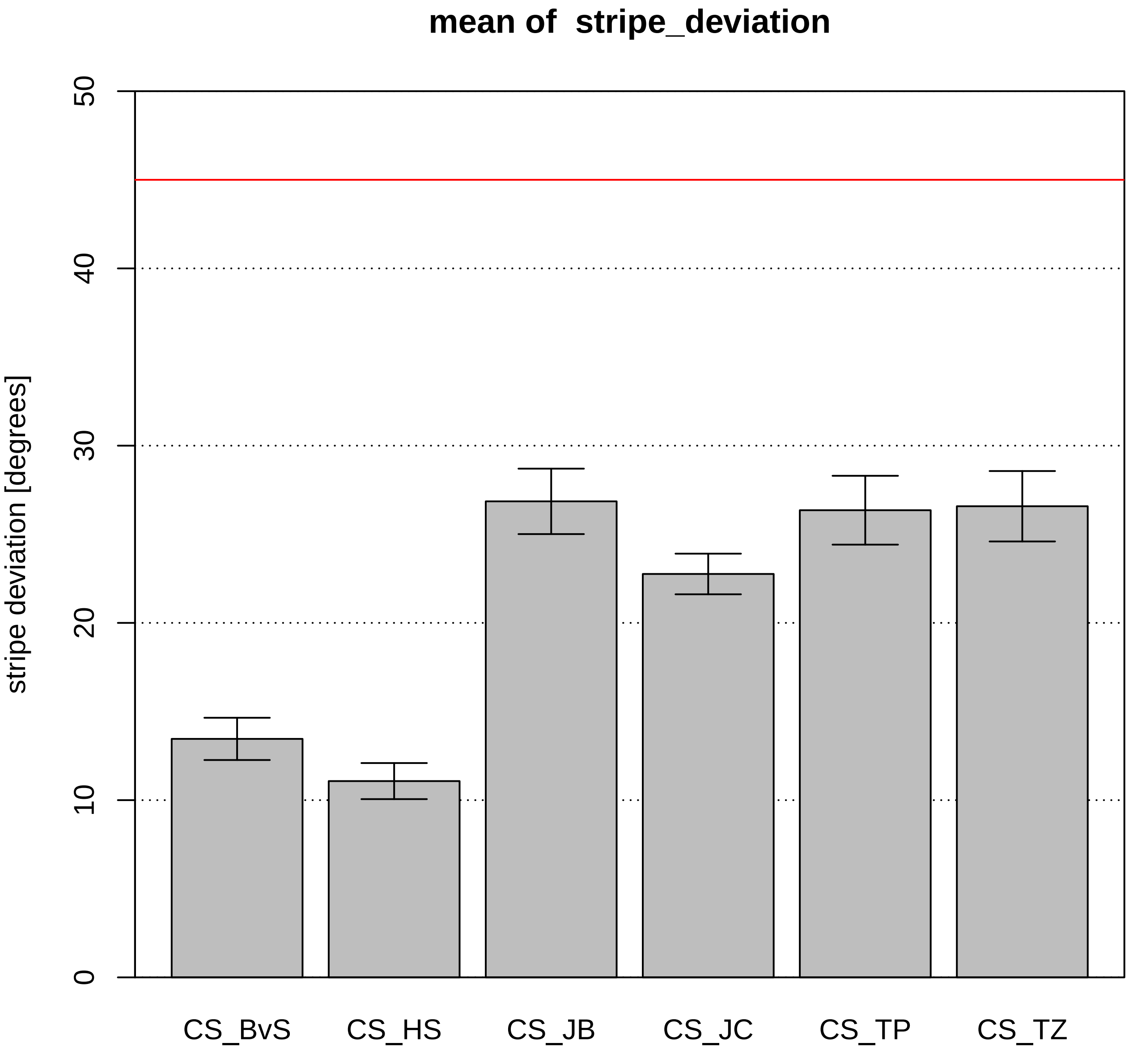

mean of stripe_deviation
stripe deviation [degrees]
0
10
20
30
40
50
CS_BvS
CS_HS
CS_JB
CS_JC
CS_TP
CS_TZ

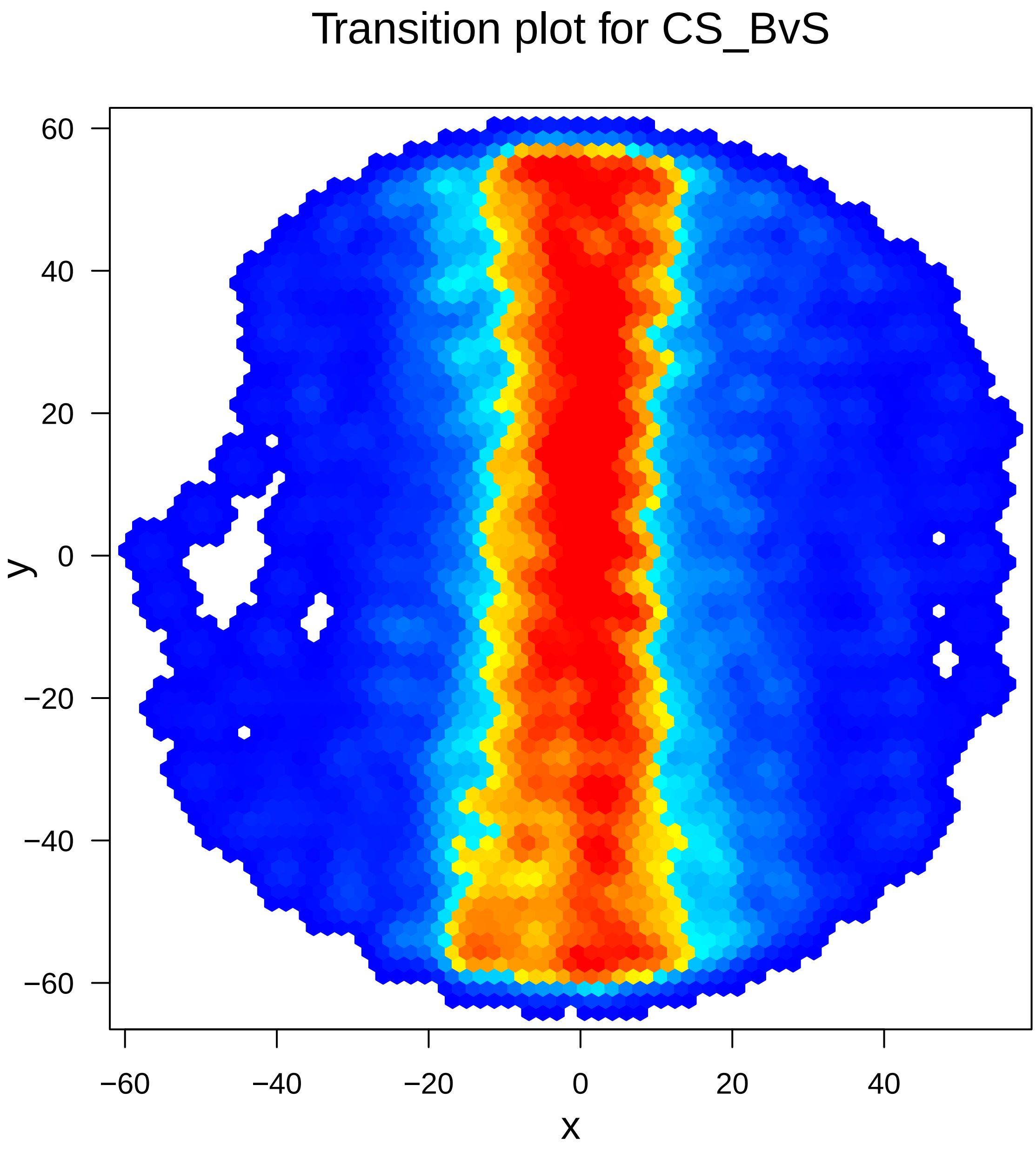
Transition plot for CS_BvS
y
x
60
40
20
0
−20
−40
−60
−60
−40
−20
0
20
40

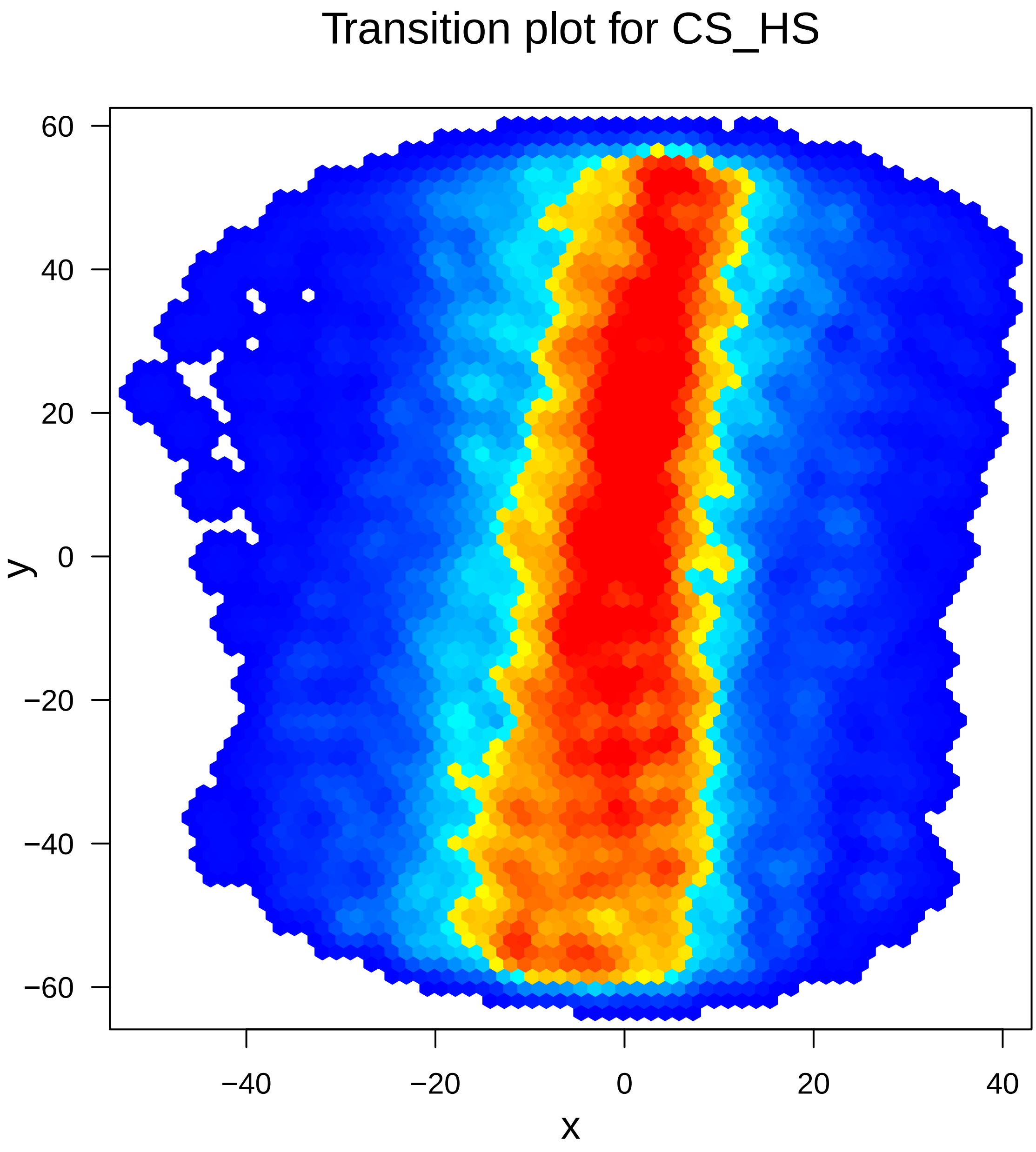
Transition plot for CS_HS
60
40
20
0
−20
−40
−60
y
−40
−20
0
20
40
x

# Transition plot for CS_JB

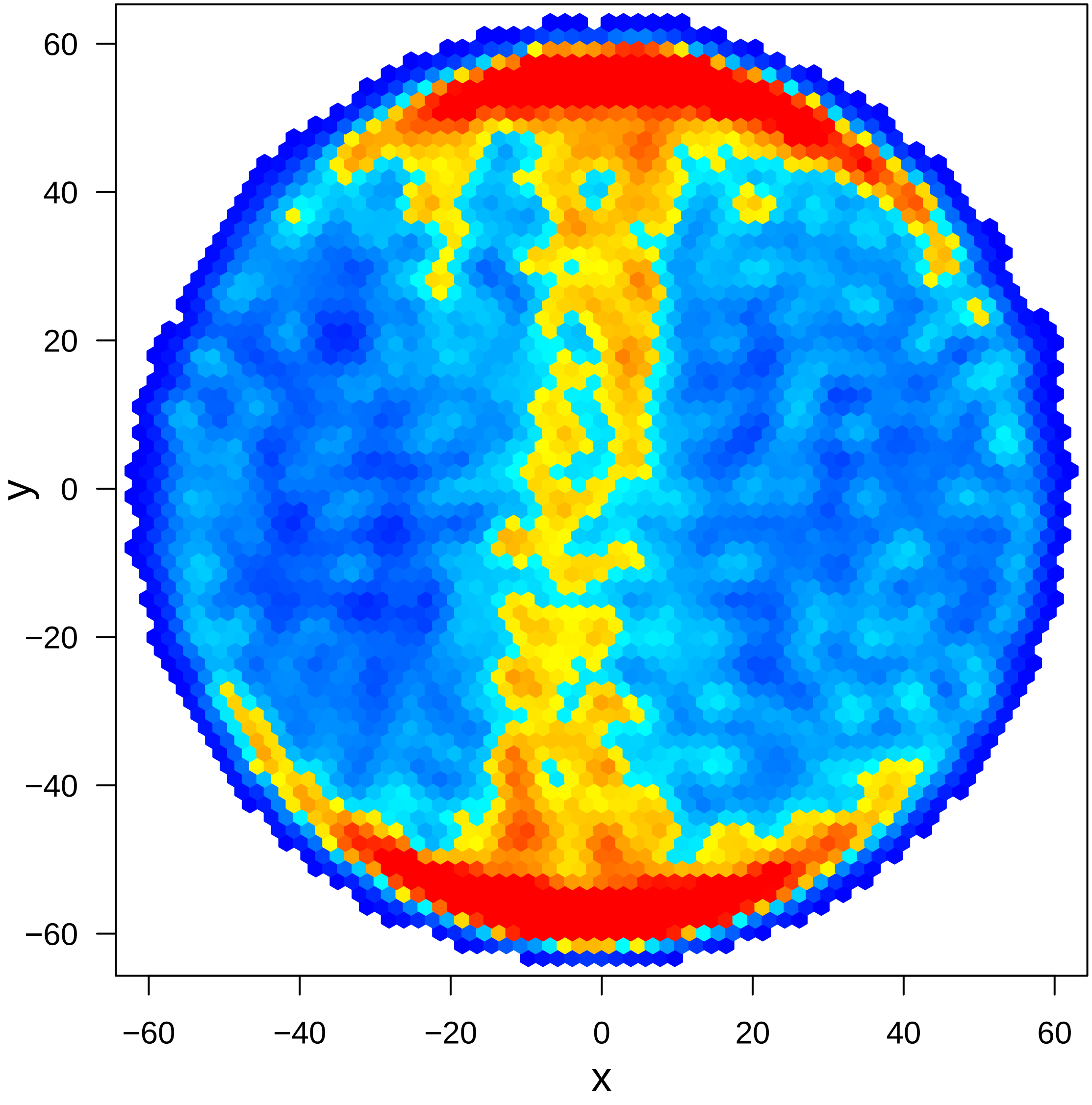

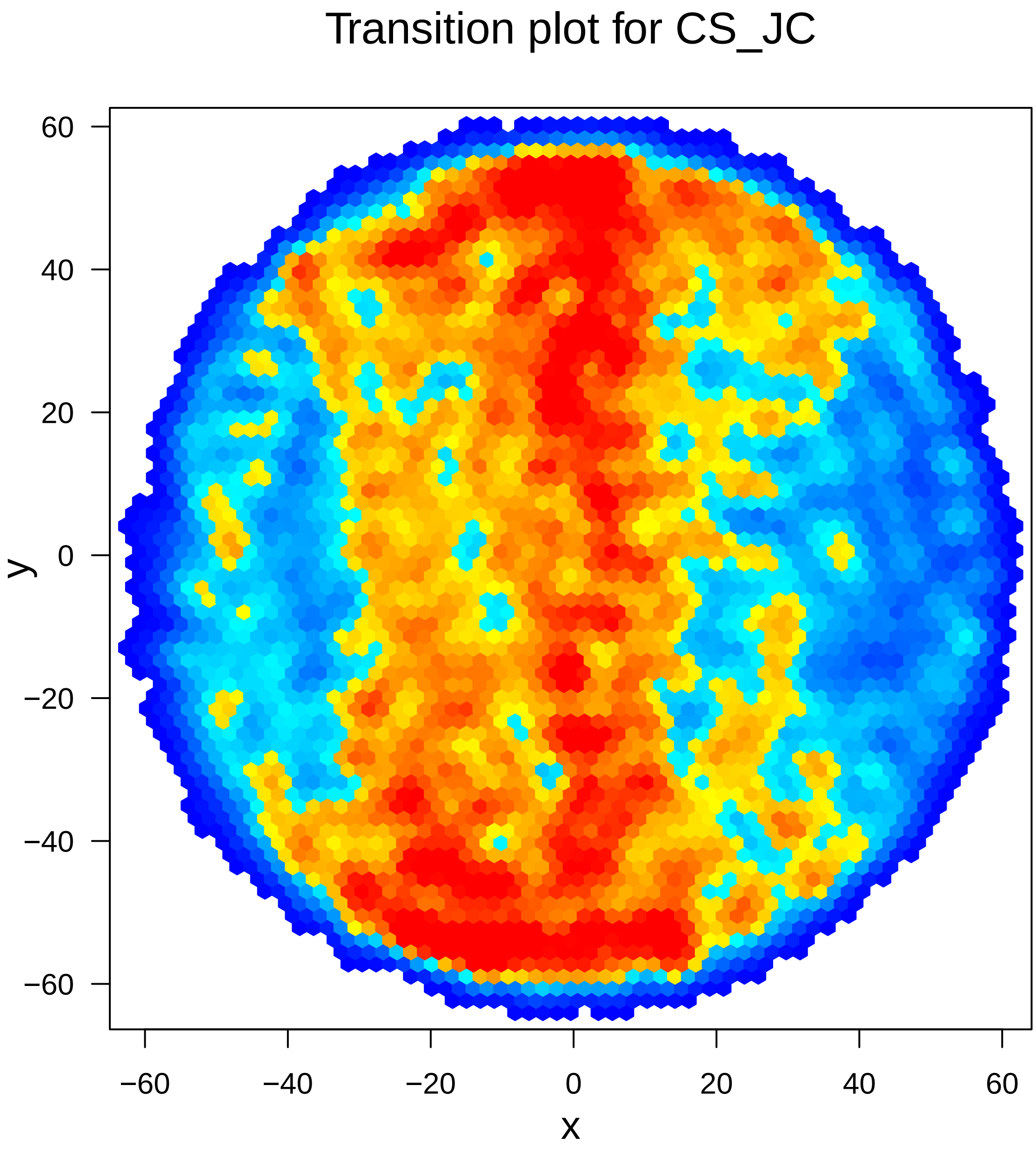

Transition plot for CS_JC
60
40
20
0
−20
−40
−60
y
−60
−40
−20
0
20
40
60
x

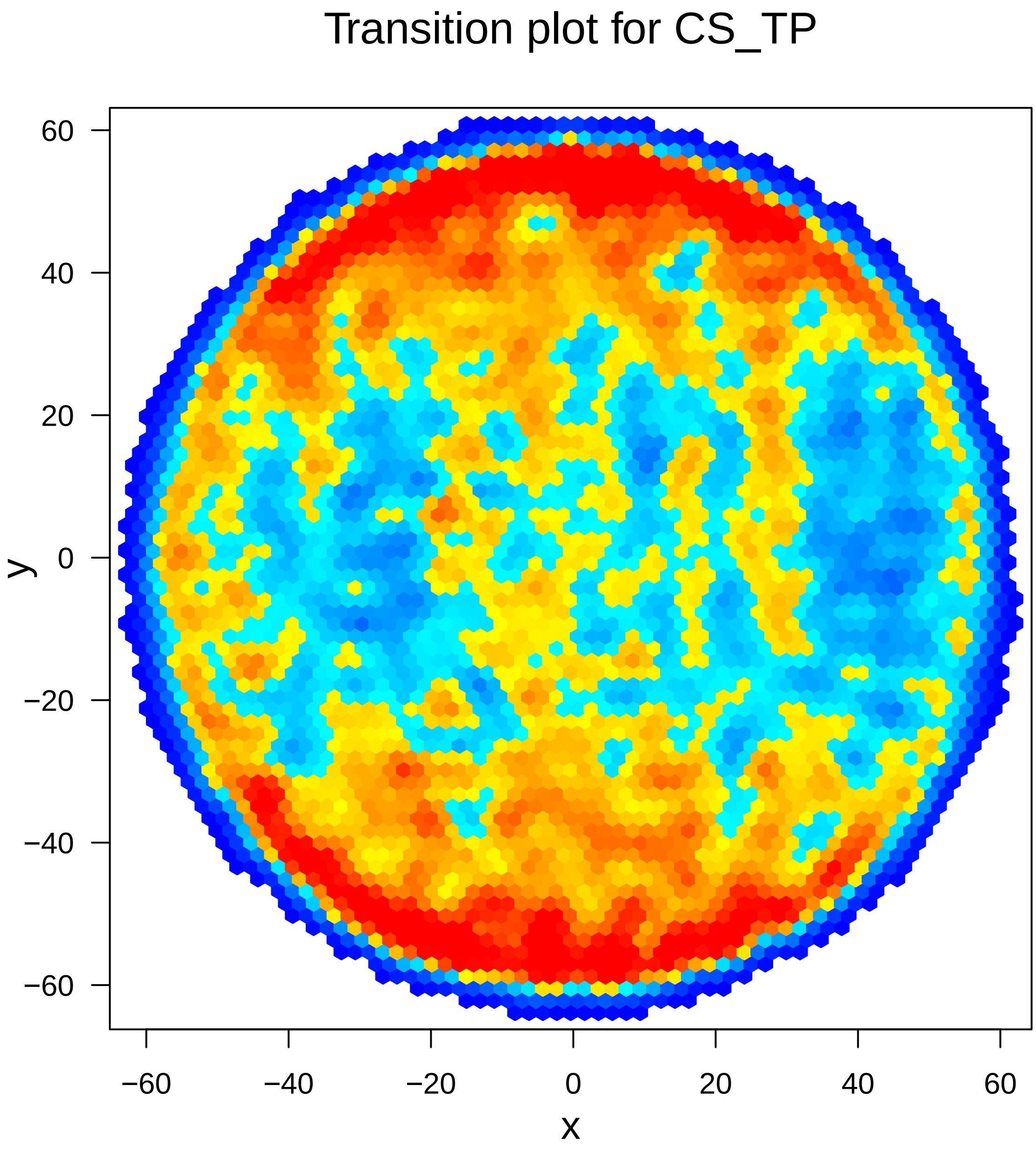

Transition plot for CS_TP
60
40
20
0
−20
−40
−60
y
−60
−40
−20
0
20
40
60
x

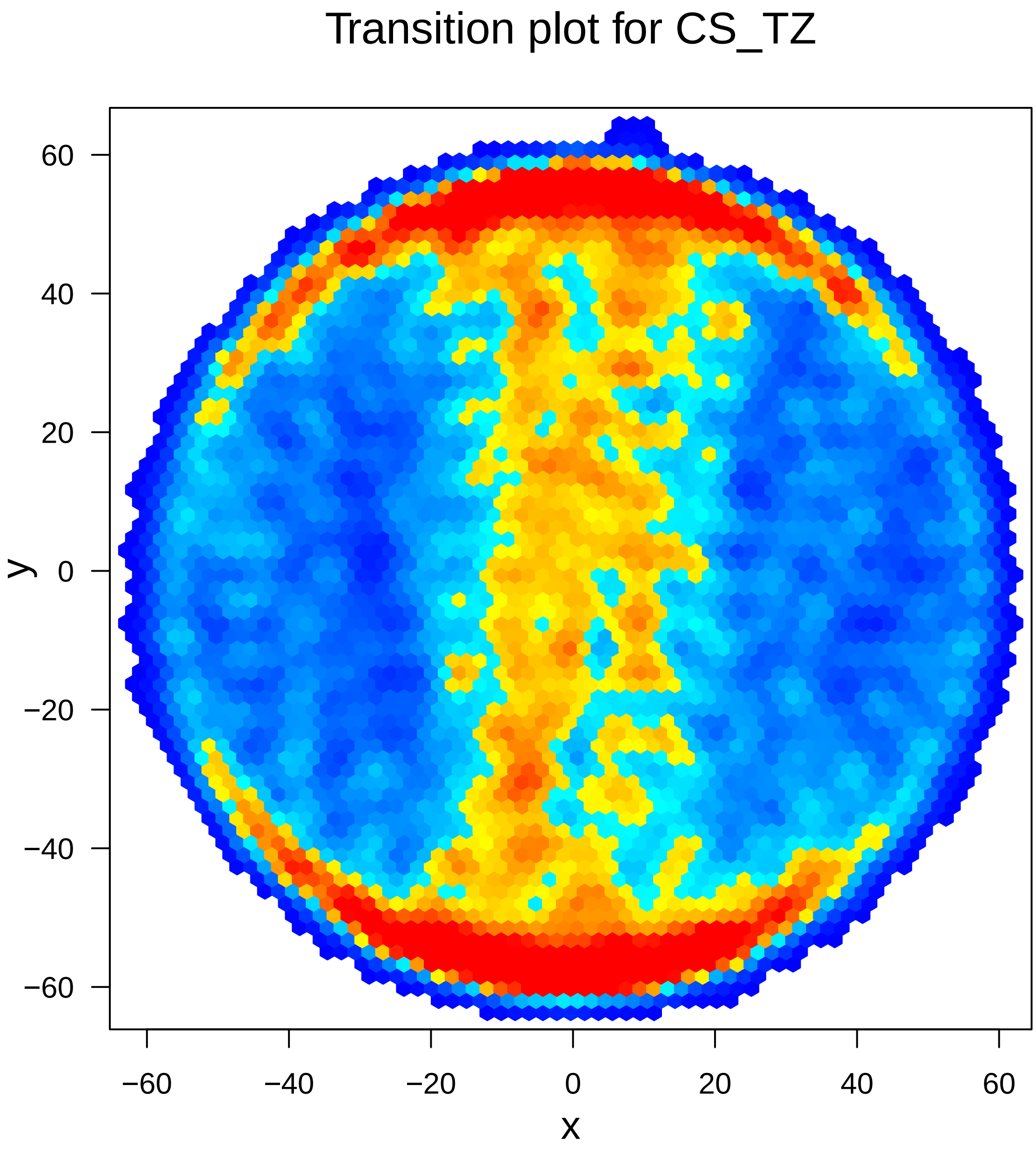

Transition plot for CS_TZ
y
60
40
20
0
−20
−40
−60
−60
−40
−20
0
20
40
60
x

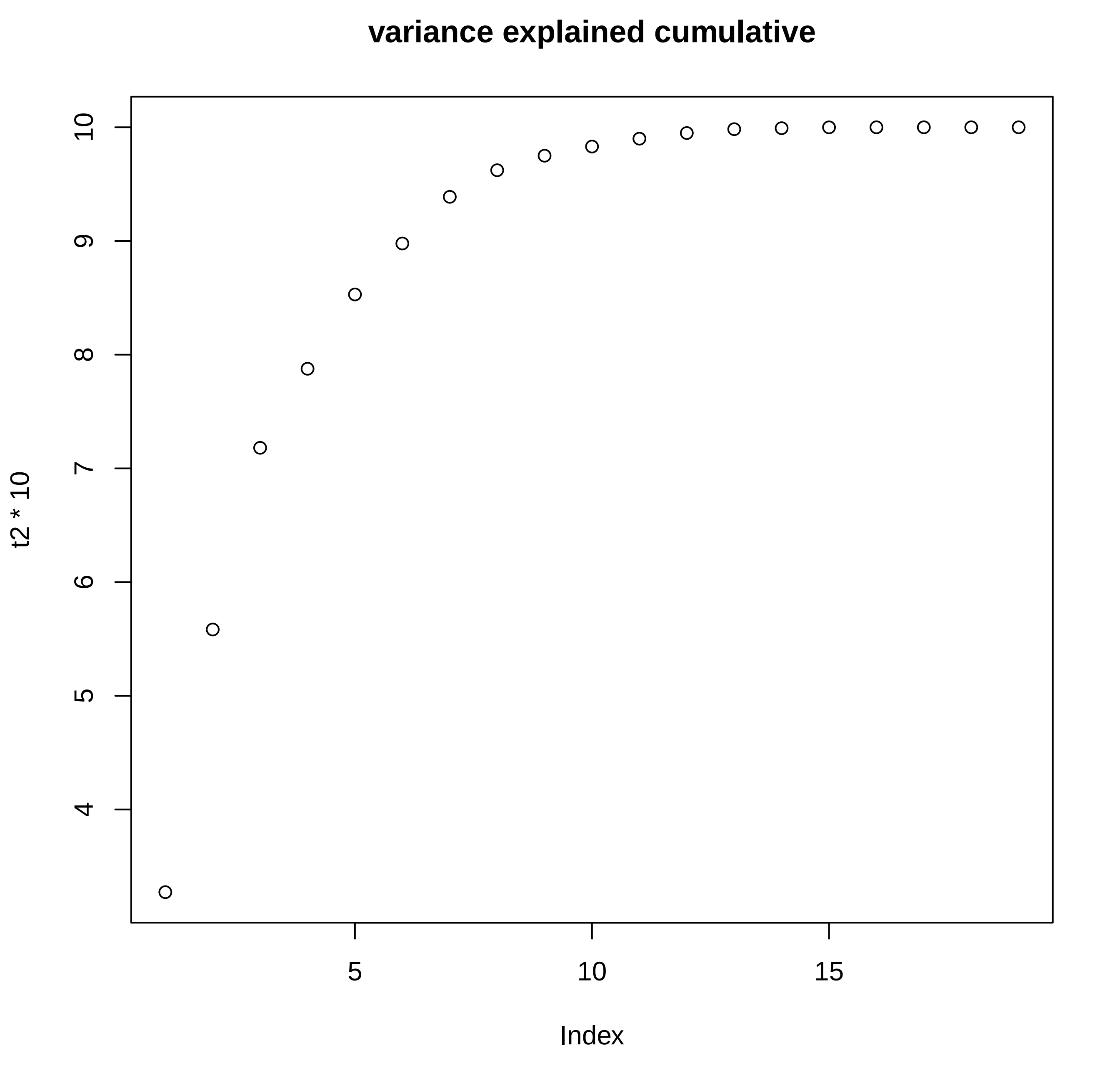
variance explained cumulative
t2 * 10
Index
4
5
6
7
8
9
10
5
10
15

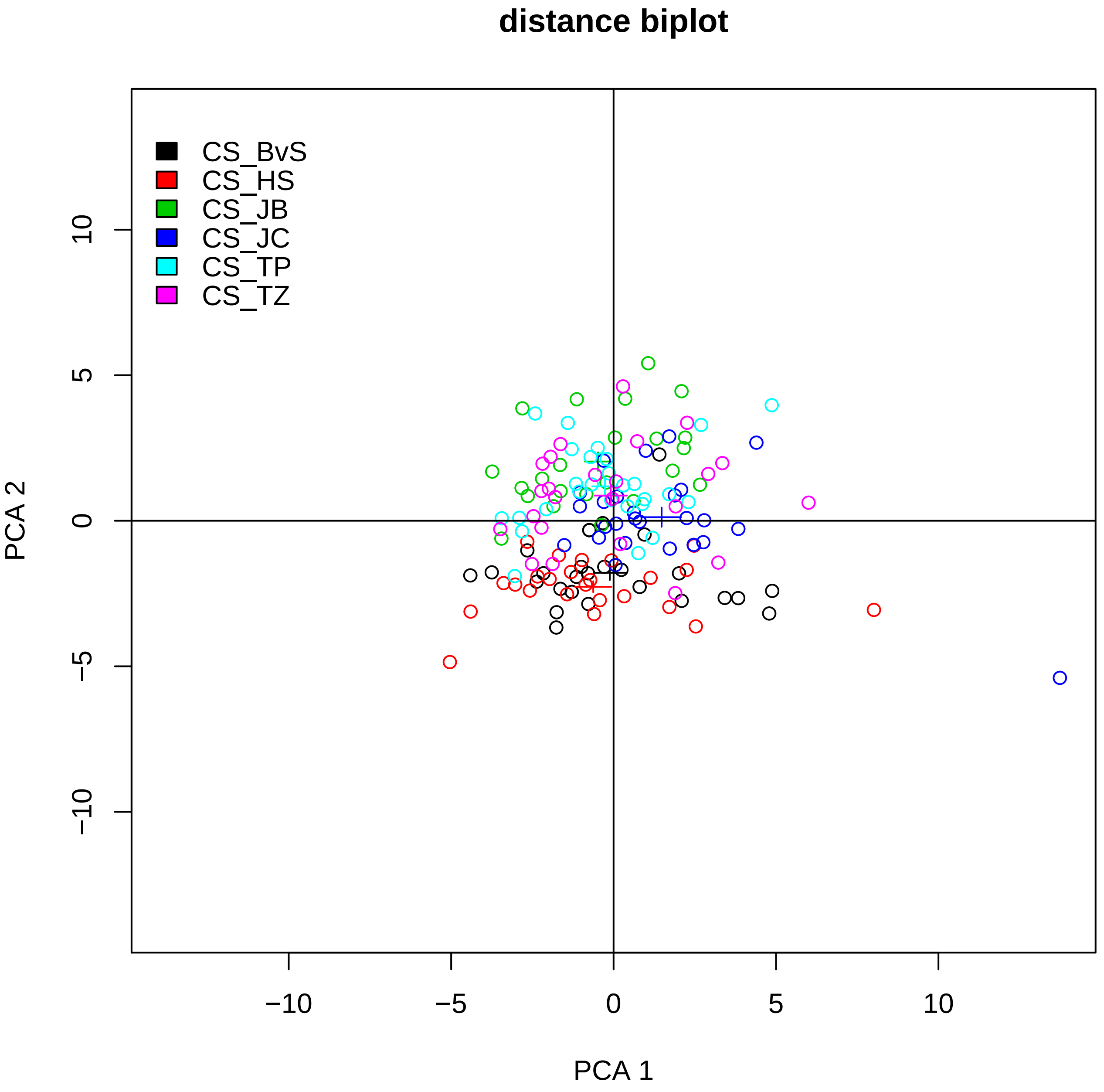

distance biplot
CS_BvS
CS_HS
CS_JB
CS_JC
CS_TP
CS_TZ
PCA 1
PCA 2
−10
−5
0
5
10

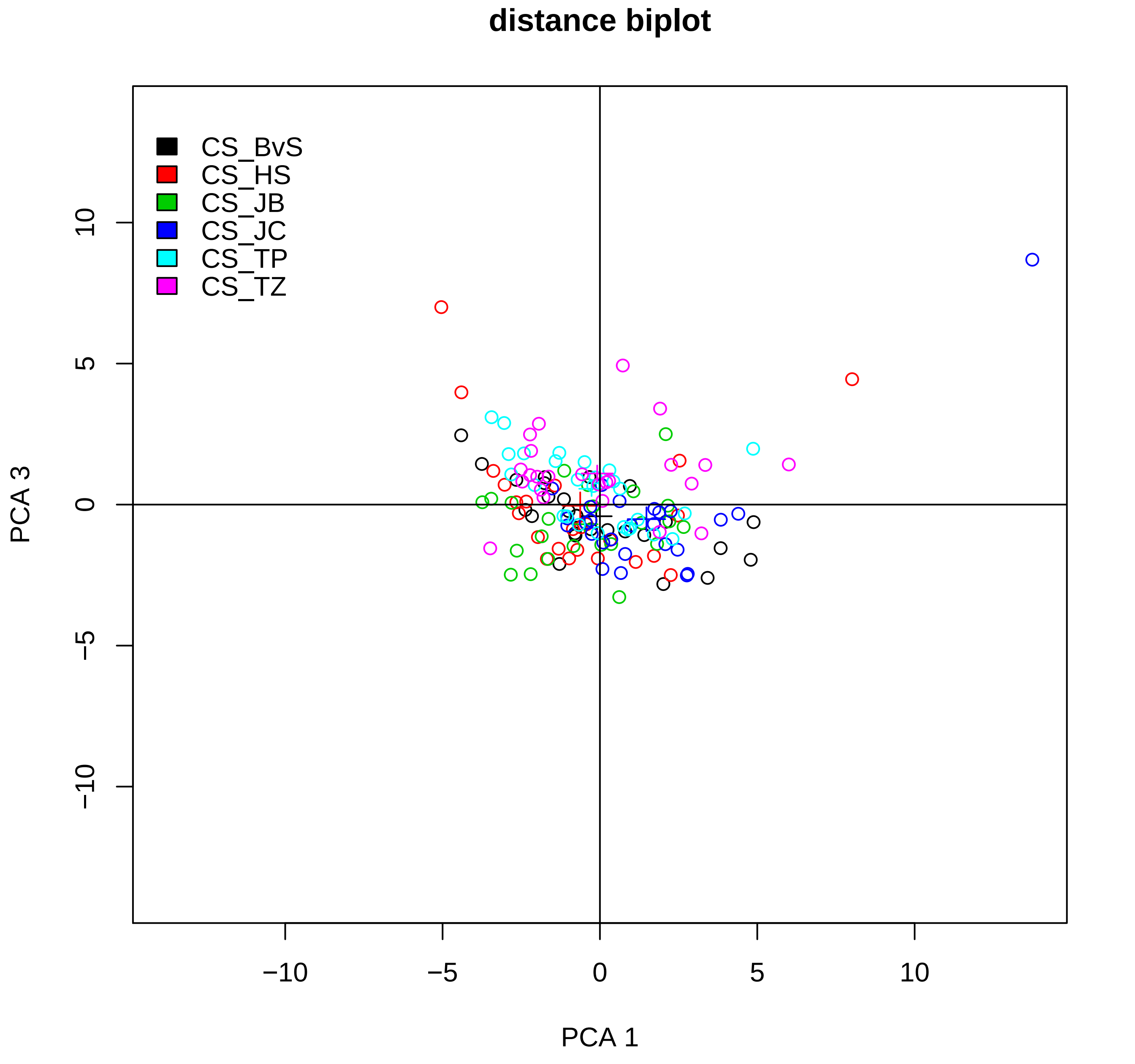

distance biplot
CS_BvS
CS_HS
CS_JB
CS_JC
CS_TP
CS_TZ
PCA 3
PCA 1
−10
−5
0
5
10

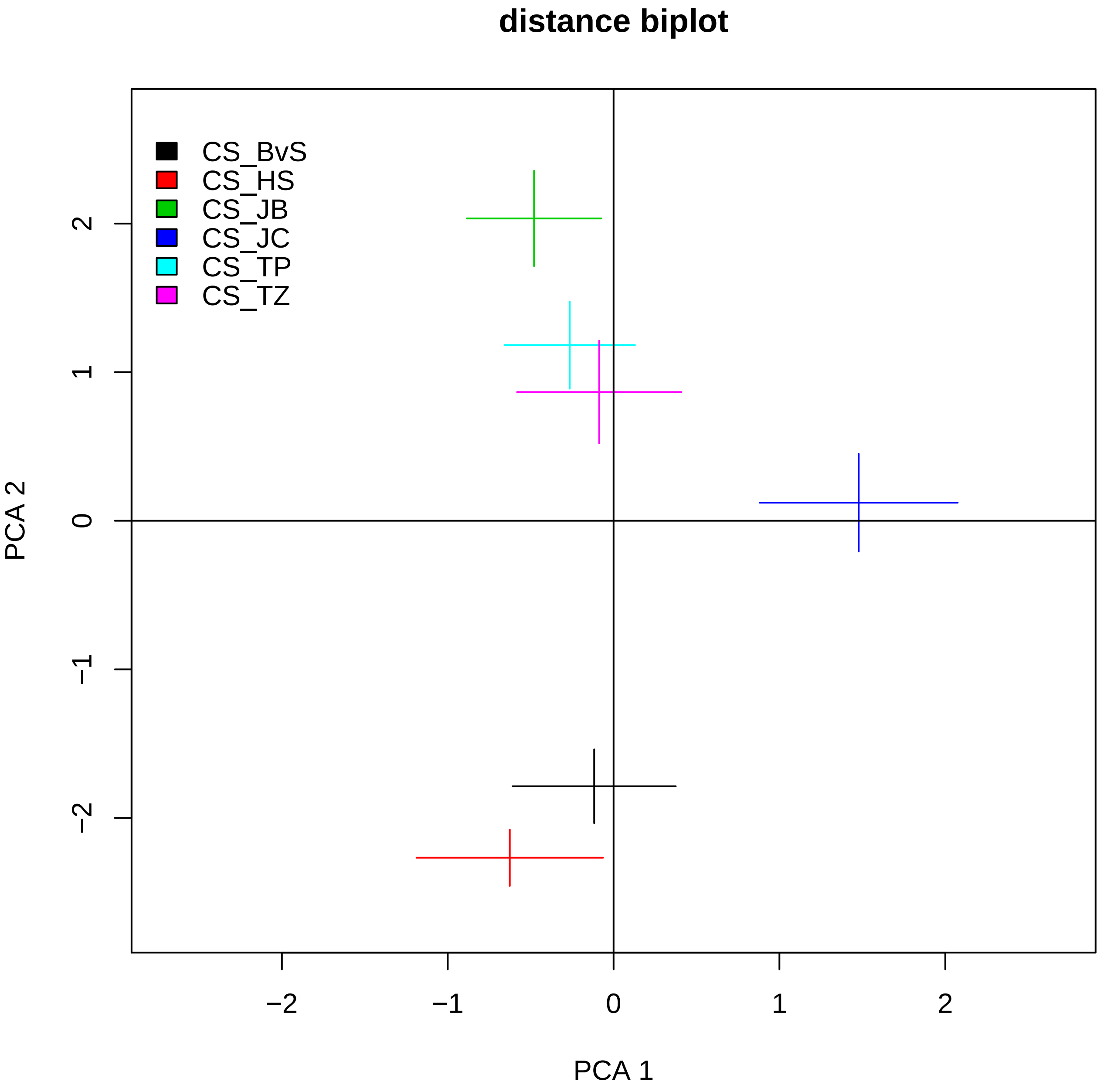
distance biplot
CS_BvS
CS_HS
CS_JB
CS_JC
CS_TP
CS_TZ
PCA 1
PCA 2
−2
−1
0
1
2

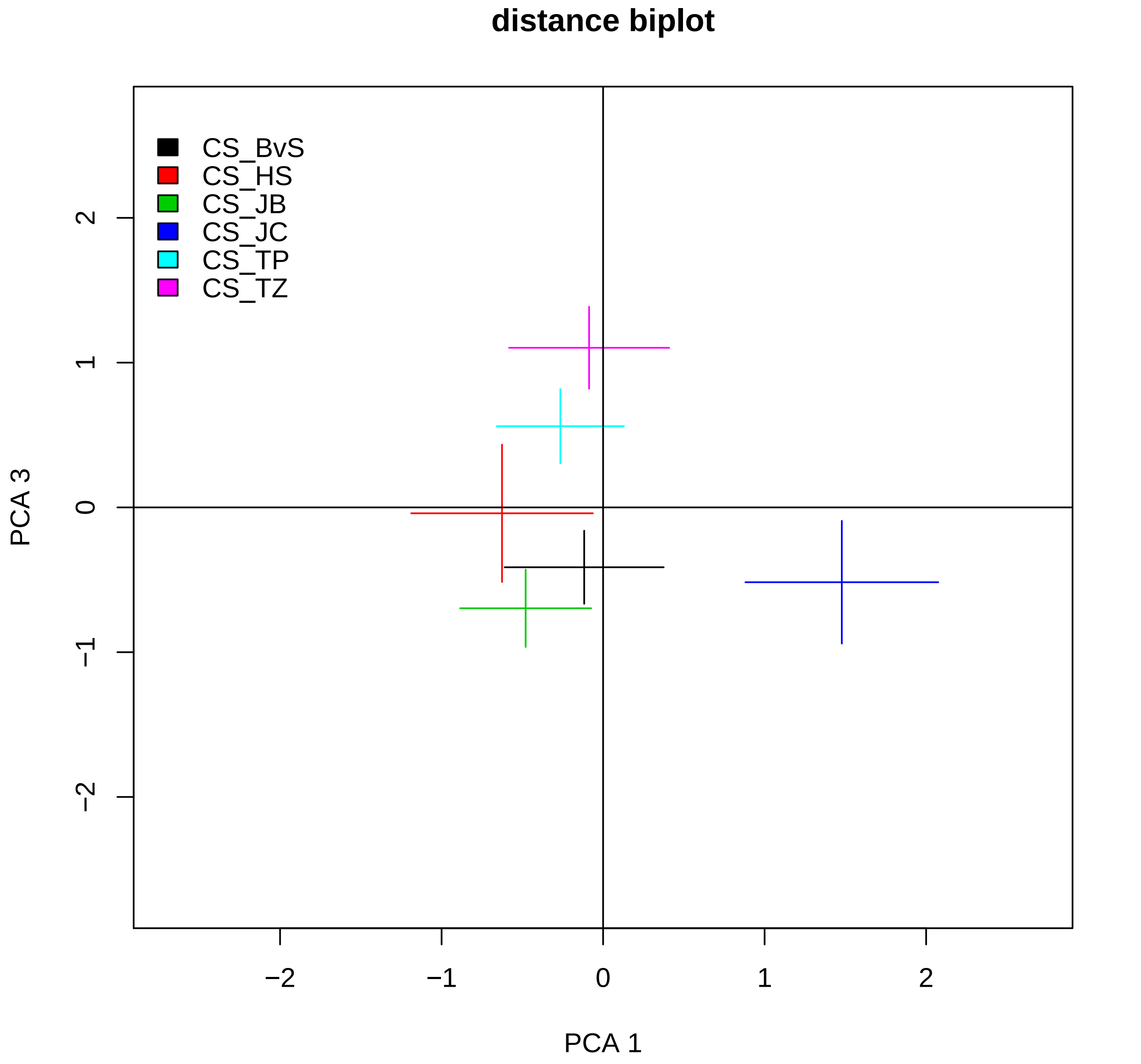
distance biplot
CS_BvS
CS_HS
CS_JB
CS_JC
CS_TP
CS_TZ
PCA 3
PCA 1
2
1
0
−1
−2
−2
−1
0
1
2

**distance biplot**

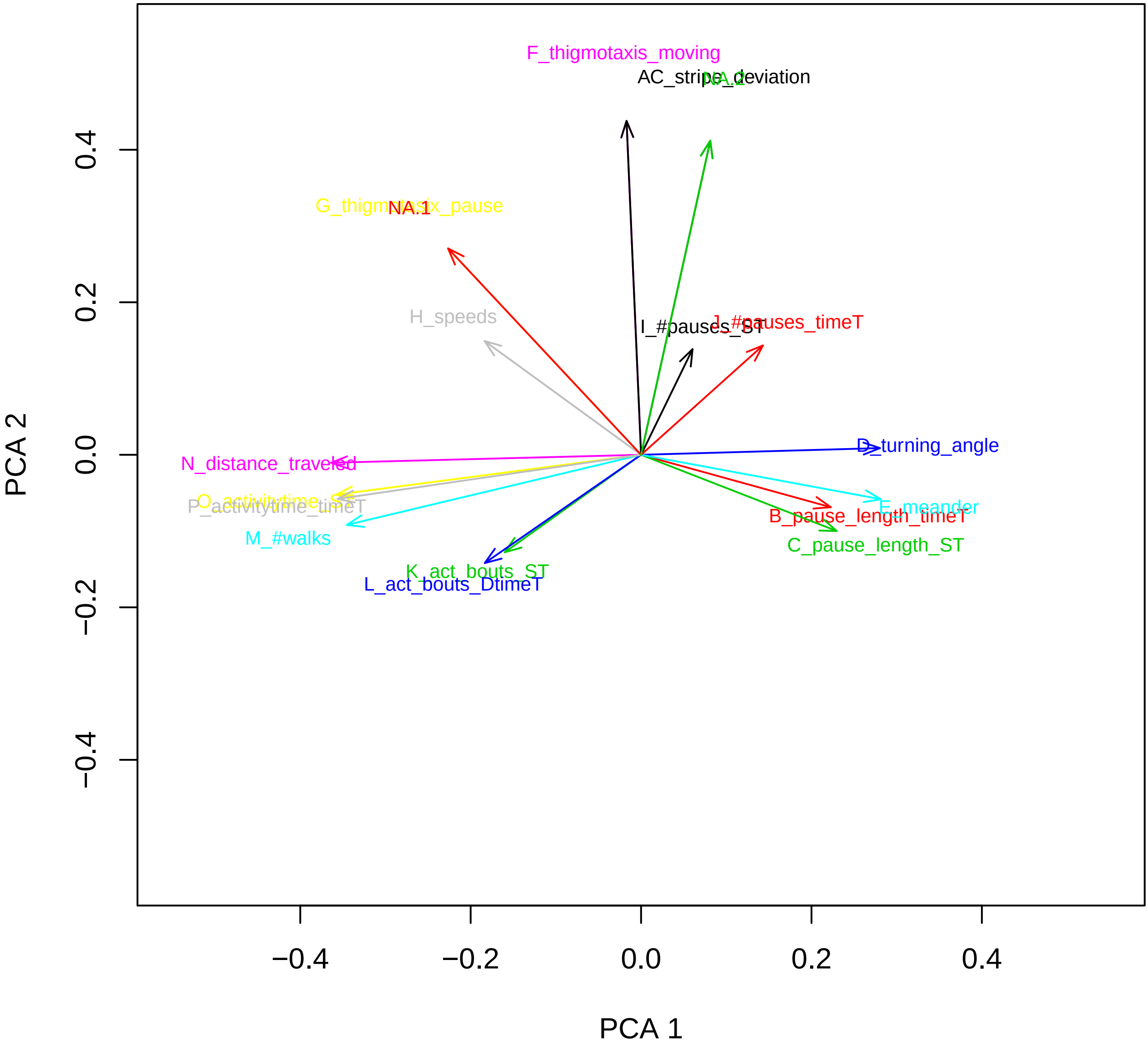

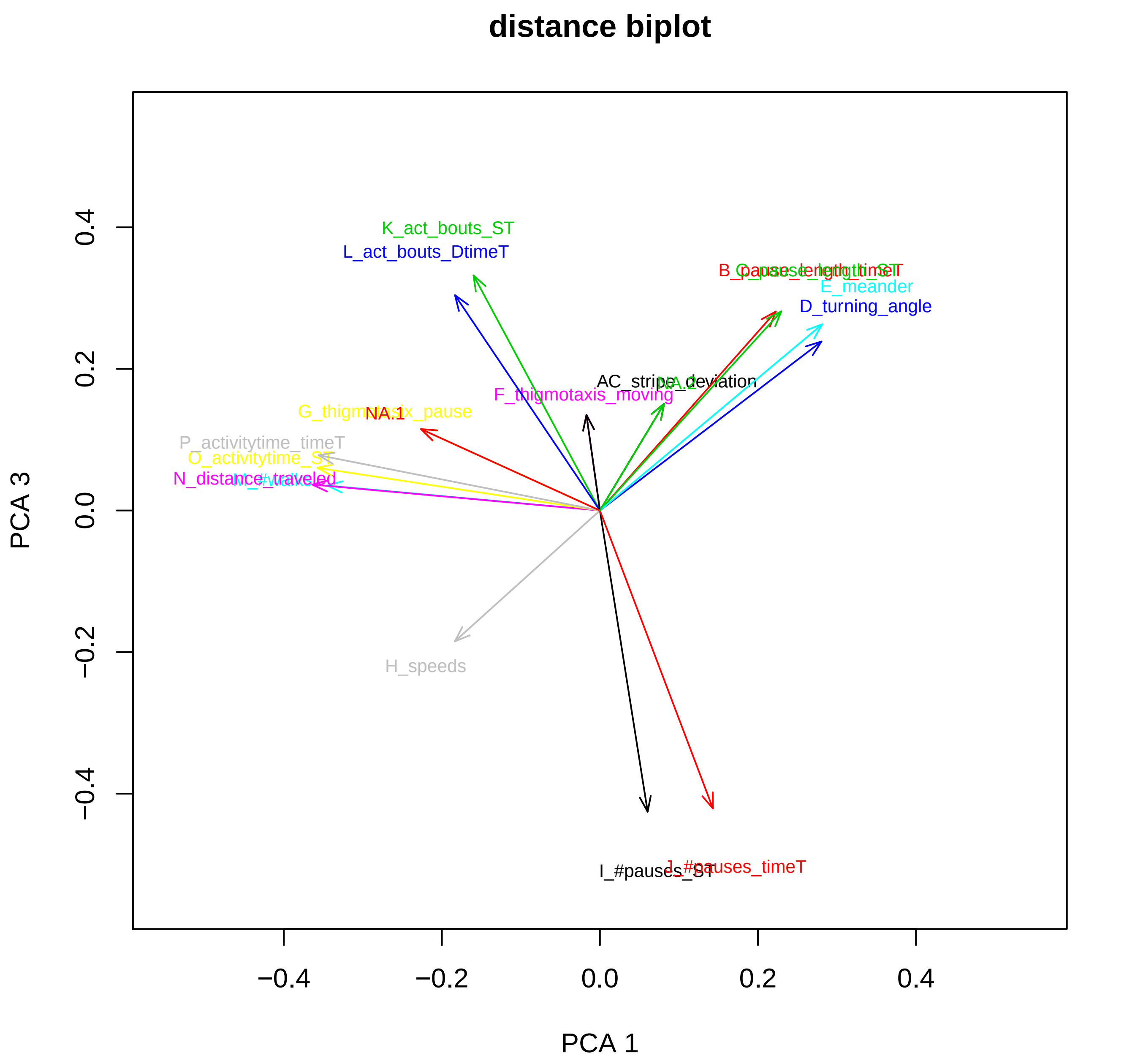
distance biplot
K_act_bouts_ST
L_act_bouts_DtimeT
E_meander
D_turning_angle
AC_stripe_deviation
F_thigmotaxis_moving
NA.1
P_activitytime_timeT
O_activitytime_ST
N_distance_traveled
H_speeds
J_#pauses_timeT
PCA 3
PCA 1
0.4
0.2
0.0
−0.2
−0.4

CS_BvS n= 27

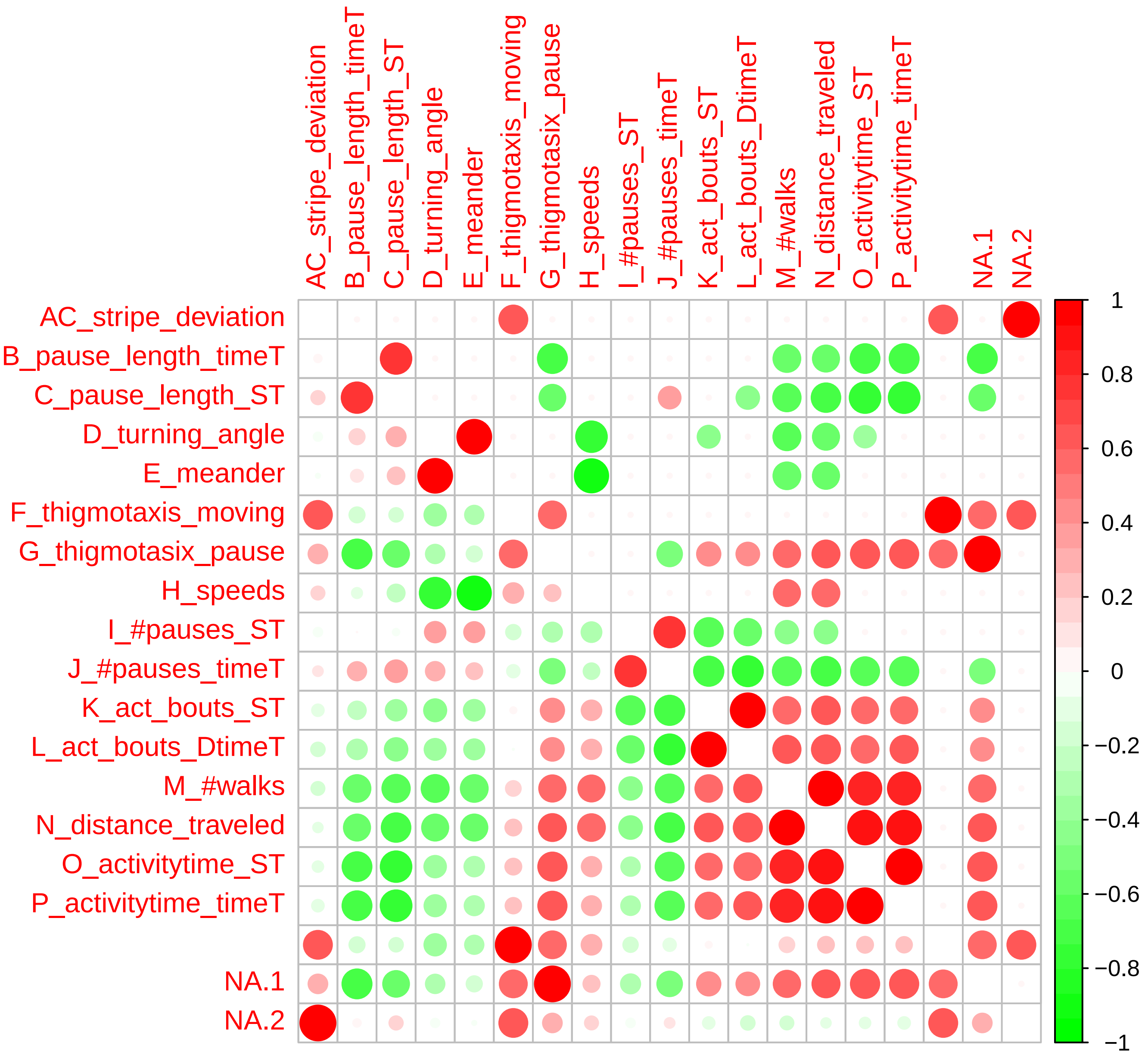
AC_stripe_deviation
B_pause_length_timeT
C_pause_length_ST
D_turning_angle
E_meander
F_thigmotaxis_moving
G_thigmotasix_pause
H_speeds
I_#pauses_ST
J_#pauses_timeT
K_act_bouts_ST
L_act_bouts_DtimeT
M_#walks
N_distance_traveled
O_activitytime_ST
P_activitytime_timeT
NA.1
NA.2
1
0.8
0.6
0.4
0.2
0
−0.2
−0.4
−0.6
−0.8
−1

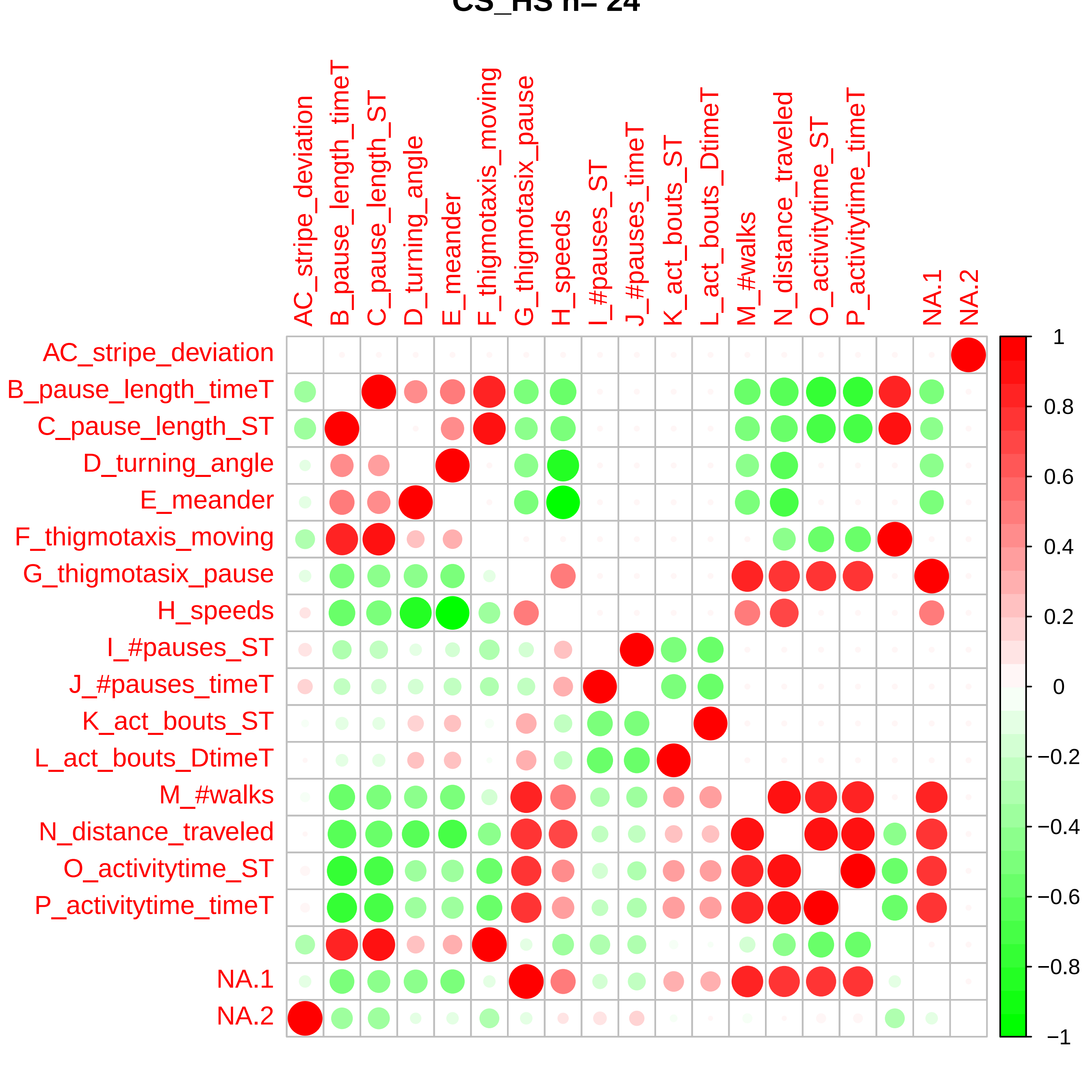

CS_HS n= 24
AC_stripe_deviation
B_pause_length_timeT
C_pause_length_ST
D_turning_angle
E_meander
F_thigmotaxis_moving
G_thigmotasix_pause
H_speeds
I_#pauses_ST
J_#pauses_timeT
K_act_bouts_ST
L_act_bouts_DtimeT
M_#walks
N_distance_traveled
O_activitytime_ST
P_activitytime_timeT
NA.1
NA.2
1
0.8
0.6
0.4
0.2
0
−0.2
−0.4
−0.6
−0.8
−1

# CS_JB n= 23

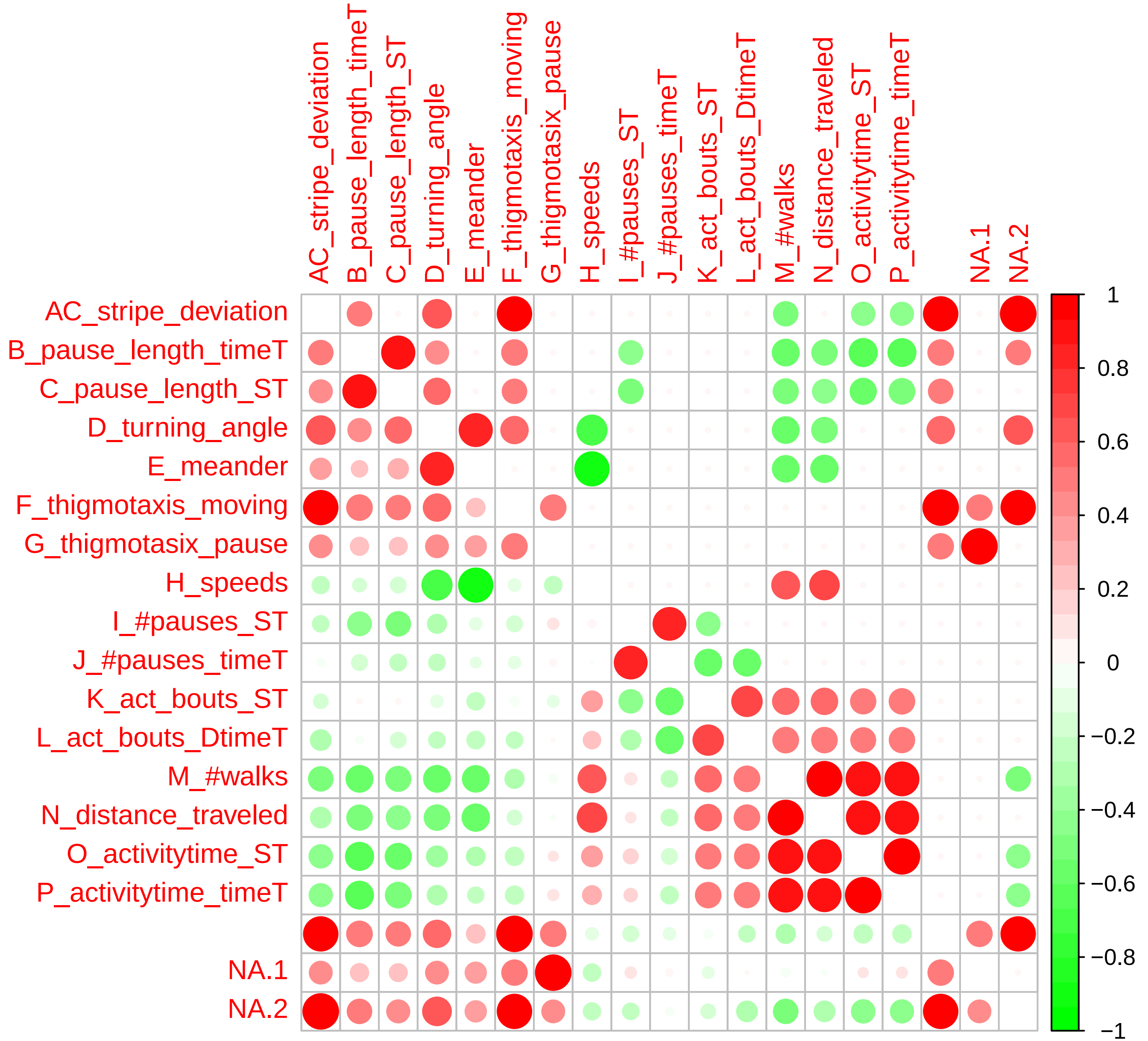
AC_stripe_deviation
B_pause_length_timeT
C_pause_length_ST
D_turning_angle
E_meander
F_thigmotaxis_moving
G_thigmotasix_pause
H_speeds
I_#pauses_ST
J_#pauses_timeT
K_act_bouts_ST
L_act_bouts_DtimeT
M_#walks
N_distance_traveled
O_activitytime_ST
P_activitytime_timeT
NA.1
NA.2
1
0.8
0.6
0.4
0.2
0
−0.2
−0.4
−0.6
−0.8
−1

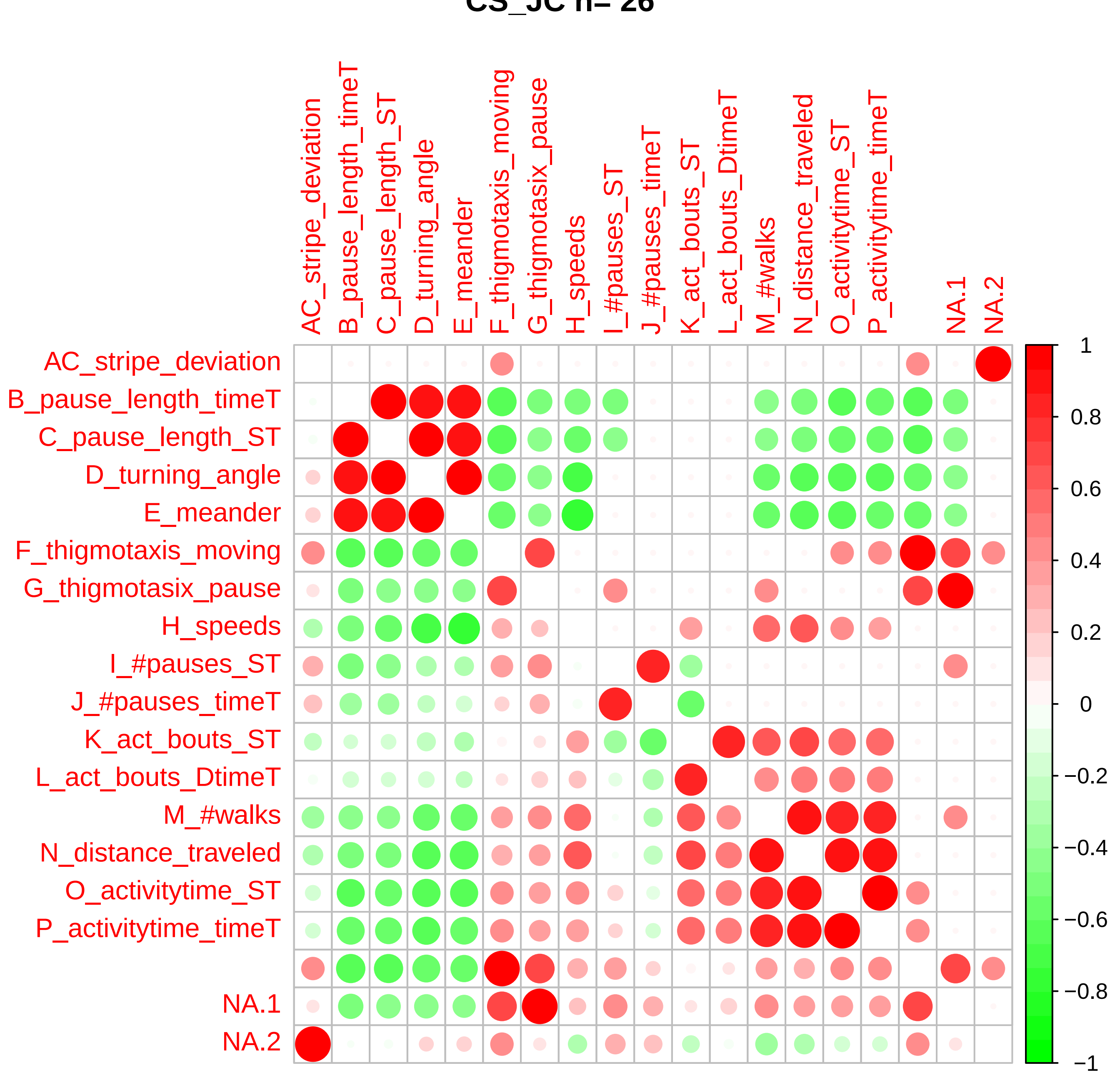
CS_JC n= 26
AC_stripe_deviation
B_pause_length_timeT
C_pause_length_ST
D_turning_angle
E_meander
F_thigmotaxis_moving
G_thigmotasix_pause
H_speeds
I_#pauses_ST
J_#pauses_timeT
K_act_bouts_ST
L_act_bouts_DtimeT
M_#walks
N_distance_traveled
O_activitytime_ST
P_activitytime_timeT
NA.1
NA.2
1
0.8
0.6
0.4
0.2
0
−0.2
−0.4
−0.6
−0.8
−1

CS_TP n= 27

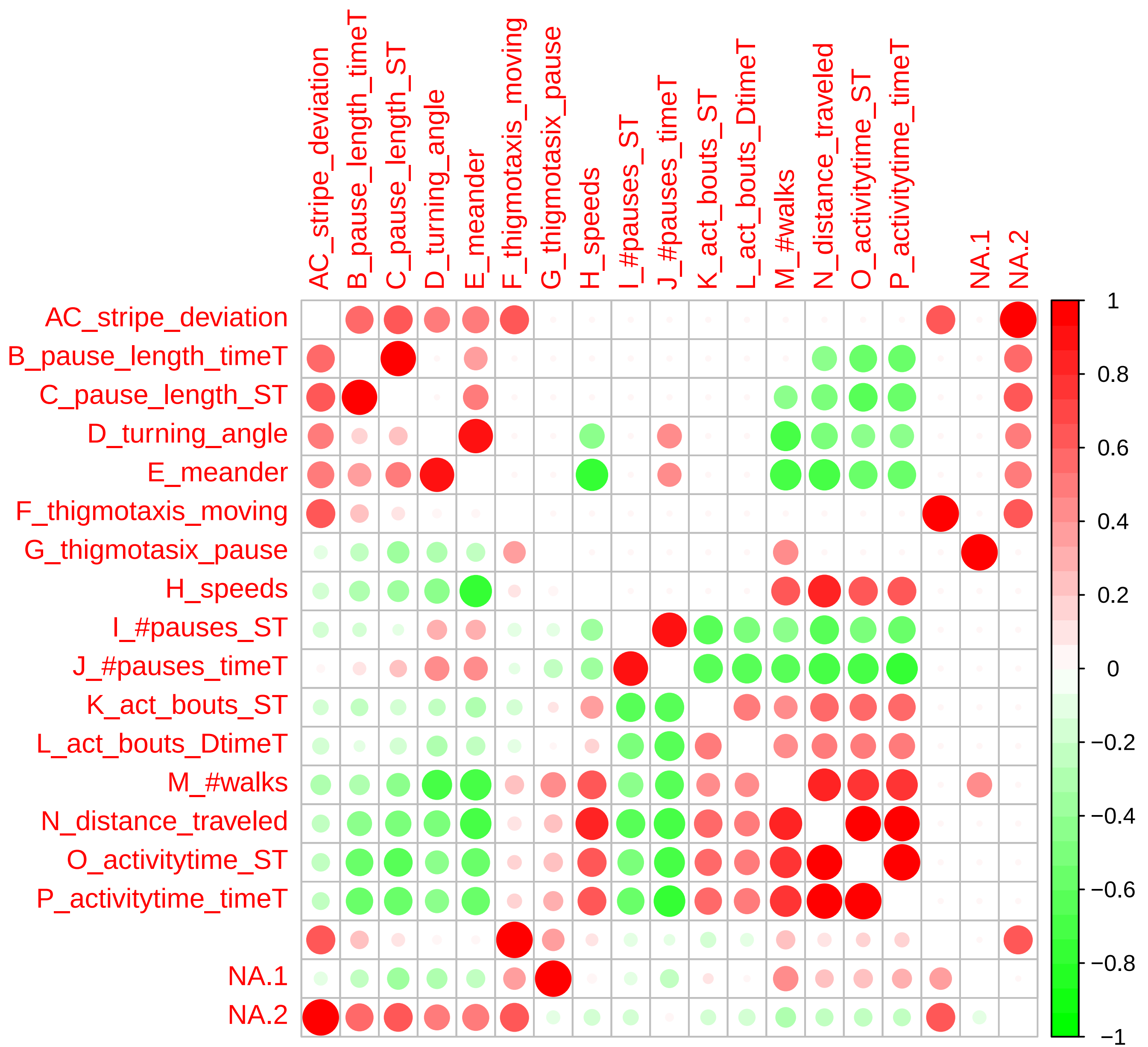
AC_stripe_deviation
B_pause_length_timeT
C_pause_length_ST
D_turning_angle
E_meander
F_thigmotaxis_moving
G_thigmotasix_pause
H_speeds
I_#pauses_ST
J_#pauses_timeT
K_act_bouts_ST
L_act_bouts_DtimeT
M_#walks
N_distance_traveled
O_activitytime_ST
P_activitytime_timeT
NA.1
NA.2
1
0.8
0.6
0.4
0.2
0
−0.2
−0.4
−0.6
−0.8
−1

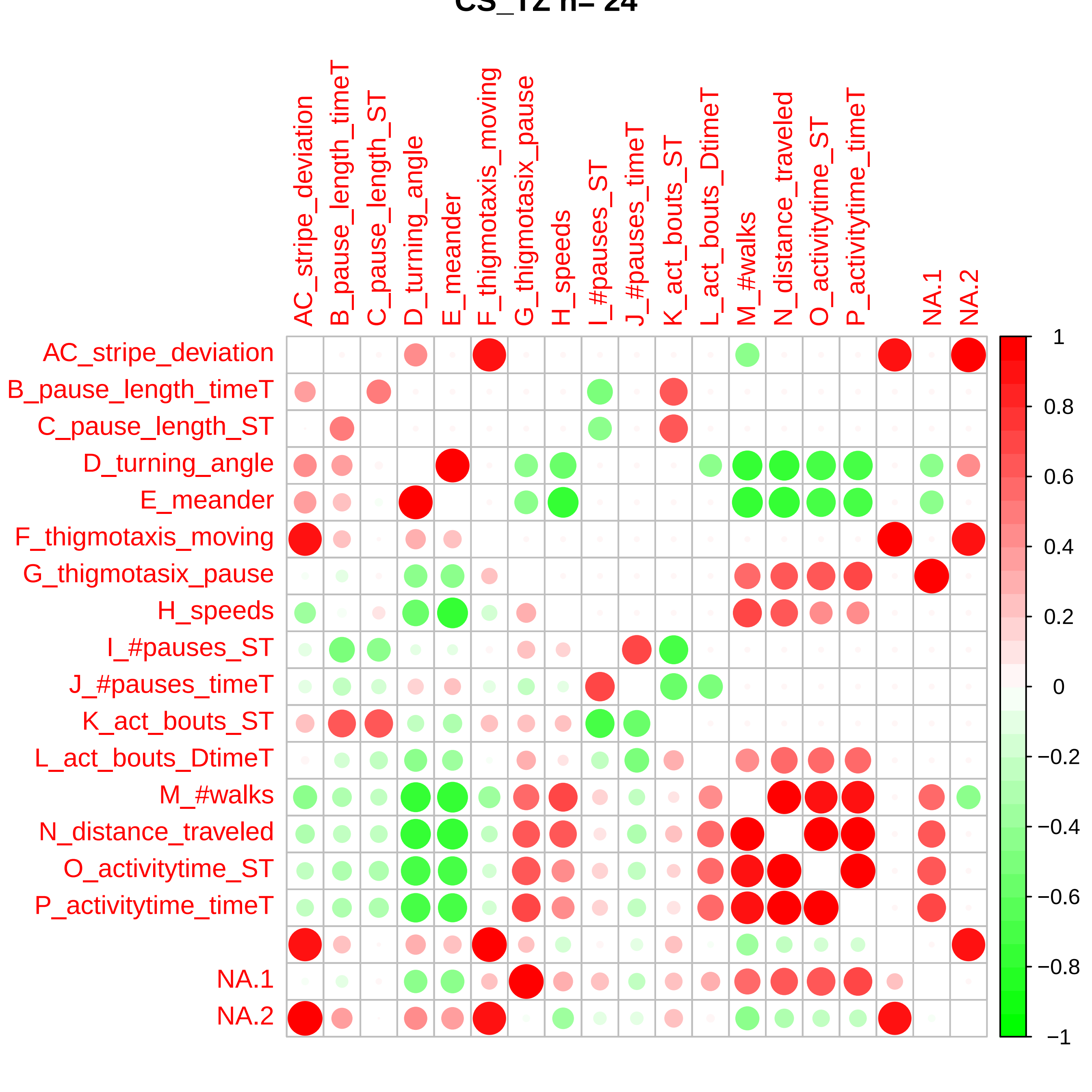
AC_stripe_deviation
B_pause_length_timeT
C_pause_length_ST
D_turning_angle
E_meander
F_thigmotaxis_moving
G_thigmotasix_pause
H_speeds
I_#pauses_ST
J_#pauses_timeT
K_act_bouts_ST
L_act_bouts_DtimeT
M_#walks
N_distance_traveled
O_activitytime_ST
P_activitytime_timeT
NA.1
NA.2
1
0.8
0.6
0.4
0.2
0
−0.2
−0.4
−0.6
−0.8
−1
