# Supplementary material for: Sub-strains of Drosophila Canton-S differ markedly in their locomotor behavior
Source: F1000Res. 2015 Apr 21;3:176. Originally published 2014 Jul 30. [Version 2] doi: 10.12688/f1000research.4263.2 (PMC4156027; doi:10.12688/f1000research.4263.2)
Supplement: Peer review history

# Peer review: Referee response for version 1

**DOI:** 10.5256/f1000research.4564.r5633

**Published:** 2014-09-02

**Authors:** Hiromu Tanimoto, Vladimiros Thoma

**Affiliations:** 1 Graduate School of Life Sciences, Tohoku University, Sendai, Japan

Colomb and Brembs reported an important piece of information that highlights large differences in the walking trajectory of six substrains of a wild-type *Drosophila* strain (Canton S) in the so-called Bridan paradigm. Drawing conclusions from behavioral comparisons of strains could therefore be moot, if the tested strains do not share the same genetic background (at least for the Buridan paradigm).

This report is presented in a clear and succinct way. In addition, the authors invite submission of data by other labs, and the addition of these new data will be plotted in Fig. 4. This is an interesting endeavor which nicely uses the function of this journal. One has yet to keep in mind that fly behavior can be dramatically affected by ‘unwritten’ lab conditions (e.g. fly food, rearing conditions), and therefore the new data from other labs might not be comparable to the current dataset. One suggestion for the contributing labs to circumvent this caveat is to use one (or more) of the strains analyzed in this study and to check the reproducibility.

In addition, I have a few minor comments that may be addressed:

1. The authors argue that the basis of the behavioral variability is differences in the genetic background, but other reasons (e.g. epigenetic differences) can conceivably contribute to the variability as well.
2. The authors state that there was a significant effect of the replicate on Principal Component 2 (page 3, left column, third line from bottom), but later state that “(…) sub-strain differences were comparable in the two replicates conducted one year apart” (page 5, left column, line 5 from top). Either of the statements should be amended.
3.  “A” in “PCA” stands for analysis. Use “PCA” instead of “PCA analysis”. My understanding of PCA is “Principal Component Analysis” rather than “Principle Component Analysis” the authors use in the paper. Use PC1, PC2, PC3 in the axes of Fig.2 and Fig. 4. 

We have read this submission. We believe that we have an appropriate level of expertise to confirm that it is of an acceptable scientific standard.

# Peer review: Referee response for version 1

**DOI:** 10.5256/f1000research.4564.r5635

**Published:** 2014-08-15

**Authors:** Josh Dubnau

**Affiliations:** 1 Cold Spring Harbor Laboratory, Cold Spring Harbor, NY, USA

This is an interesting manuscript that I hope will be read widely.  In this field, we give a lot of lip service to controlling genetic background because we all know it is important.  But often, "wild type" lines such as CS are assumed to be equivalent and this turns out to be quite problematic conceptually.  Now Colomb and Brembs have put some empirical evidence forward that confirms our fears about this practice.  CS is a commonly used outbred strain, but my CS and your CS are different due to founder effects, drift, and selection.  In this particular study, the founder effect has a massive impact on Buridan's paradigm, a relatively simple locomotor behavior.  And this would likely be true for any quantitative trait. 

I have one comment on the conclusion; the effects seem to be mostly due to founder effects rather than selection (drift should be considered as well).  This seems like a likely explanation in this current comparison, but this might change when we deal with mutants and transgenes maintained on an outbred strain.  Such mutants or transgenes may cause accumulation of modifiers, which I would consider to be selection based.

Regardless of the underlying mechanism, I like the fact that the authors of this study propose a solution, in which a stock center might maintain a large population of an outbred strain such as CS, and then we all would outcross our mutants and transgenes to that line.  This could work, and offers the advantage that results from different groups would be comparable.  Without such a mechanism, it is important, at a minimum, that all labs at least back cross all mutants/transgenes to their own CS or equivalent w.t. line.  On the other hand, I would argue that genetic screens and collections of large populations of Gal4 and other such lines should be generated on an INBRED background which eliminates or reduces founder effects.  This provides a more useful reagent for the community to use because it eliminates the onerous need to outcross every single line from a large collection.

Overall, this is an important issue, and this study does a nice job of shedding light with actual data.

I have read this submission. I believe that I have an appropriate level of expertise to confirm that it is of an acceptable scientific standard.

# Peer review: Referee response for version 1

**DOI:** 10.5256/f1000research.4564.r5630

**Published:** 2014-08-08

**Authors:** Gregg Roman

**Affiliations:** 1 Department of Biology and Biochemistry, University of Houston, Houston, TX, USA

This manuscript does an excellent job demonstrating significant strain differences in Burdian's paradigm. Since each Drosophila lab has their own wild type (usually Canton-S) isolate, this issue of strain differences is actually a very important one for between lab reproducibility. This work is a good reminder for all geneticists to pay attention to the population effects in the background controls, and presumably the mutant lines we are comparing.  

I was very pleased to see the within-isolate behavior was consistent in replicate experiments one year apart. The authors further argue that the between-isolate differences in behavior arise from a Founder's effect, at least in the differences in locomotor behavior between the Paris lines CS_TP and CS_JC. I believe this is a very reasonable and testable hypothesis. It predicts that genetic variability for these traits exist within the populations. It should now be possible to perform selection experiments from the original CS_TP population to replicate the founding event and estimate the heritability of these traits.  

Two other  things that I liked about this manuscript are the ability to adjust parameters in figure 3, and our ability to download the raw data. After reading the manuscript, I was a little disappointed that the performance of the five strains in each 12 behavioral variables weren't broken down individually in a table or figure. I thought this may help us readers understand what the principle components were representing. The authors have made this data readily accessible in a downloadable spreadsheet.

I have read this submission. I believe that I have an appropriate level of expertise to confirm that it is of an acceptable scientific standard.
